# Explainable Ensemble Learning With Stain Normalization and Deep Feature Extraction for Acute Lymphoblastic Leukaemia Classification

**DOI:** 10.1049/htl2.70091

**Published:** 2026-07-14

**Authors:** Faysal Ahmmed, Md. Sadi Al Huda, Md. Asraf Ali, M. M. Manjurul Islam

**Affiliations:** ^1^ Department of Computer Science and Engineering American International University‐Bangladesh (AIUB) Dhaka Bangladesh; ^2^ Department of Computer Science and Engineering Khwaja Yunus Ali University Sirajganj Bangladesh; ^3^ Khwaja Yunus Ali University Sirajganj Bangladesh; ^4^ Ulster University Coleraine UK

**Keywords:** acute lymphoblastic leukaemia (ALL), B‐cell ALL (B‐ALL), deep neural network, image processing, machine learning, peripheral blood smear images, stacking ensemble learning, Vahadane, VGG16

## Abstract

Acute lymphoblastic leukaemia (ALL), a common form of cancer, remains a life‐threatening condition that affects individuals worldwide, including both adults and children. The prognosis is significantly worse when the disease is identified at an advanced stage, as the chances of successful treatment diminish. Diagnosis typically requires invasive and costly procedures, which can delay timely intervention. Images from peripheral blood smears (PBS) have been useful for the preliminary screening of ALL in probable cases. However, due to the nonspecific presentation of the disease, interpreting these images poses significant challenges, increasing the risk of misdiagnosis. We have proposed an approach that uses 20,000 PBS images to precisely enable the early diagnosis of ALL and its distinct subtypes (benign, malignant‐early Pro‐B, malignant‐Pro‐B, and malignant‐Pre‐B) in order to overcome these problems. Our proposed system uses colour normalized PBS images using Vahadane method along with the feature extraction capabilities of deep neural networks combined with a stacking ensemble learning approach that integrates various machine learning algorithms for classification. This system is capable of distinguishing ALL cases from haematogones and accurately identifying its subtypes. By streamlining the diagnostic process, the platform aims to reduce the effort and time required by clinicians and patients. Our proposed model highlight the system's effectiveness, with a comparative analysis of established machine learning algorithms showcasing its superior performance. The proposed model achieved exceptional accuracy (99.95%), recall (99.95%), precision (99.95%) and F1‐score (99.95%) for the early detection of ALL and its variants, indicating its potential as a proof‐of‐concept system, while requiring further validation before clinical deployment.

## Graphical abstract

We propose an automated framework for early detection of acute lymphoblastic leukaemia (ALL) and its subtypes using 20,000 peripheral blood smear images. The method combines Vahadane‐based colour normalization, deep neural network feature extraction, and a stacking ensemble classifier to accurately distinguish ALL from haematogones and identify disease subtypes. Experimental results demonstrate superior performance, achieving 99.95% accuracy, precision, recall and F1‐score, thereby reducing diagnostic effort and time.

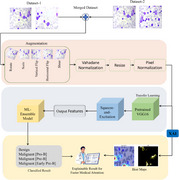

## 1 Introduction

Acute lymphoblastic leukaemia (ALL), also known as lymphocytic leukaemia, is a type of cancer caused by immature lymphocytes in bone marrow [1]. There are two types of acute lymphocytic leukaemia disease. Among them about 85% is B‐cell ALL (B‐ALL) [2] type which results from lymphoid blood cell cancer, that can be identified by bone marrow lymphocytes, a particular type of white blood cells, that proliferate rapidly [3]. These dysfunctional lymphocytes compromise the immune system and interfere with the ability of the bone marrow to produce essential components such as platelets and red blood cells. A single immature or blast cell can produce billions of other blast cells [4].

There were 64,200 ALL cases globally in 2017 compared to 49,100 in 2017 [5]. B‐ALL was responsible for 437,033 reported cases and 303,006 related deaths globally in 2022, according to research published by the International Agency for Cancer Research of the World Health Organization (WHO) and the International Agency for Research on Cancer (IARC) [6].

Early clssification of B‐ALL is crucial, as late‐stage delays or diagnoses are often associated with higher mortality rates [7]. However, early diagnosis remains a persistent challenge for haematologists, oncologists and researchers [8]. B‐ALL is frequently associated with symptoms such as enlarged liver and spleen, pallor, fever, bone discomfort, chronic infections, easy bruising and significant weight loss. However, these symptoms are nonspecific and can overlap with other medical conditions, making early diagnosis particularly difficult [9]. There are a number of diagnostic methods and specialized tools [10] are needed to classify B‐ALL. Such as cytogenetic analysis, Immunophenotyping, bone marrow aspiration, and lumbar puncture [11] can lead to significant complications, especially in paediatric patients, including pain, bruising and bleeding [9]. Additionally, patients are financially burdened by the high expense of bone marrow tests, especially those who need numerous samples. rural areas often face challenges such as limited access to healthcare facilities, shortages of specialized medical professionals and financial constraints, which impede timely cancer diagnosis and treatment [12]. Traditional manual analysis techniques, like peripheral blood smear (PBS) assessment, are time‐consuming, labour‐intensive and prone to errors. Intervention efforts may become less effective or unfeasible due to diagnostic errors or delays brought on by the tedious manual screening of PBS samples. On the other hand, PBS samples can be frequently examined under a microscope for the fast screening of B‐ALL using a robust clinical applicable system which can reduce the mortality rates.

Nowadays, machine learning techniques are helpful in determining the presence of ALL and the classification of B‐ALL in medical applications [13]. Transfer learning is a method for applying a previously trained machine learning model to a new dataset while presuming that the original model's prejudiced abilities would remain useful. Most high‐performing models have already undergone a thorough training phase, therefore adapting to a new dataset will likely take less time. For the same reasons, fewer data points will be required to fine‐tune the model on a target dataset, hence large data sets may not be required to achieve optimal performance. Several freely available pre‐trained machine learning models for a variety of purposes have undergone rigorous evaluation and benchmarking [14].

To overcome these challenges, this study proposed a set of machine learning and deep learning approaches for classifying ALL and the type of B‐ALL disease at an early stage. The proposed approaches combine the computational efficiency of conventional machine learning techniques for classification with deep neural networks (DNNs) for feature extraction. In particular, high‐dimensional, meaningful features are extracted from colour‐normalized microscopic pictures using pre‐trained convolutional neural network (CNN) architectures, VGG16, which greatly reduces the requirement for manually created feature engineering and processing overhead. The best individual model for performance is then identified by feeding these extracted features into a variety of machine‐learning classifiers. To further enhance diagnostic accuracy and robustness, this study integrates these classifiers into an ensemble learning model. The ensemble approach aggregates the strengths of multiple classifiers to deliver superior diagnostic outcomes, ensuring reliability and scalability in clinical applications. Furthermore, we deployed a prototype website using SREAMLIT, which allows users to upload PBS images. Once the images are uploaded, the model automatically classifies the ALL subtype, providing users with real‐time results directly through this platform. The primary contributions of this research are as follows:

- A unified framework is introduced that combines dataset merging, multi operation data augmentation, and Vahadane stain normalization to address data scarcity, class imbalance and colour inconsistency in peripheral blood smear images, resulting in a more stable and representative input space.
- An extensive evaluation of stain normalization techniques, including Macenko, Vahadane, and hematoxylin–eosin (H&E) separation, is conducted. Deep discriminative features are extracted using VGG16, and these normalized features are utilized to train multiple machine learning classifiers for accurate detection of ALL and its subtypes.
- Multiple machine learning classifiers such as SVM, random forest and XGBoost are trained and compared using VGG16 extracted features. These models are further combined into a stacking ensemble classifier, which achieves the highest performance and demonstrates improved robustness across all ALL subtypes.
- Interpretability is enhanced through the integration of Grad CAM++ for visual explanations and LIME for feature level insights. In addition, a real time Streamlit based prototype is developed, enabling users to upload PBS images and obtain instant diagnostic predictions.

To address the above contributions, this study explores the following research questions:

RQ1: How can stain normalization techniques, such as Macenko, Vahadane and H&E, improve the consistency and diagnostic accuracy of ALL and B‐ALL subtype classification?

RQ2: How effectively can pre‐trained CNN models (VGG16) serve as feature extractors for peripheral blood smear images?

RQ3: Which machine learning algorithms and ensemble strategies provide the most robust and reliable performance for classifying ALL and its subtypes using deep CNN‐generated features?

RQ4: How do data augmentation and dataset merging strategies help mitigate class imbalance and data scarcity in ALL subtype classification?

RQ5: How can interpretability techniques such as Grad‐CAM++ and LIME improve the transparency, interpretability, and clinical trustworthiness of automated ALL diagnostic models?

RQ6: How can a real‐time diagnostic system be designed to support early ALL screening and ensure practical usability in low‐resource or clinical environments?

The rest of the paper is organized as follows: a literature review is given in Section 2, the methodology is described in Section 3, results and findings are in Section 4 along with limitations, and finally, a conclusion and recommendation are summarized in Section 5.

## 2 Literature Review

In order to diagnose ALL, Sampathila et al. [3] presented an innovative network architecture called ALLNET. A publicly accessible microscopic image dataset, the ALL Challenge dataset from ISBI 2019, was used to train and evaluate this architecture. Their findings showed that ALLNET's impressive 95.54% accuracy rate indicated its potential for accurately detecting ALL. A crucial component of thorough disease care, the study did not address the categorization of leukaemia cells into distinct subtypes.

Using blood smear images from the AA‐IDB2 database, a hybrid architecture for leukaemia detection [15] was developed. It utilizes cutting‐edge optimization and segmentation strategies. A hybrid model that used mutual information (MI), fuzzy C‐means, and active contour approaches were utilized during the segmentation phase. Important characteristics including colour histograms and local difference patterns (LDP) were then taken out and utilized as inputs for a CNN architecture that was based on sine cosine aAlgorithm (SCA). The SCA algorithm improved the leukaemia detection accuracy by optimizing the CNN's weights. Though the framework presented a robust methodology for identifying leukamia in blood smear images, it required significant domain expertise for effective utilization, especially for detecting ALL in single‐cell images. Moreover, the study did not focus on classifying leukaemia into its subtypes.

Studies examining the effects of different optimization strategies for hyper‐parameter tuning in deep learning models customized for ALL diagnosis are severely lacking, according to the corpus of current research. In order to detect ALL in microscopic blood smear images, a convolutional neural network was proposed [16] that was improved using Bayesian techniques. In order to customize the CNN architecture and its parameters to the input data, this method made use of Bayesian optimization, an iterative procedure that minimizes an objective error function by modifying the hyper‐parameter space. A hybrid dataset created by combining two publicly available datasets was used to train and evaluate the model. The hybrid dataset was improved by this data augmentation method, which also helped the model perform better. In comparison to previous deep learning optimized models, the experimental results showed that the Bayesian‐optimized CNN detected ALL from blood smear images with more accuracy. However, the study demonstrated the efficacy of this approach for ALL classification, but it failed to extend its scope to include the identification of specific ALL subtypes. This limitation is significant, as accurate diagnosis and effective treatment planning depend on correctly identifying the subtype and the disease's progression within the body.

In another study, using the YOLO v4 object detection algorithm [17] introduced an automated technique for identifying ALL blast cells in peripheral blood smear images. The method used was trained and evaluated using two publicly available datasets, C_NMC_2019 and ALL‐IDB1, which contain 10,769 preprocessed single‐cell pictures. On both the C_NMC_2019 and ALL‐IDB1 datasets, the model's mean average precision (MAP) was 98.7% and 96.06%, respectively. Experimental results showed that after 6,000 iterations, the loss decreased significantly, stabC_NMC_2019 and ALL‐IDB1ilizing at approximately 0.57664. Despite the method's strong performance, the study left room for further improvement in diagnostic accuracy and robustness. This opens avenues for frameworks, such as the one proposed in this study, to enhance performance and achieve even higher reliability in ALL detection.

In 2022, a study [18] introduced a deep learning‐based approach for identifying ALL and its subtypes using PBS images. A collection of 3256 PBS pictures taken from 89 people suspected of being affected by ALL was used in the study. For analysis, the system used paired segmented and original images and included a cost‐conscious cell segmentation process. Their model employed a DenseNet‐201 architecture for feature extraction, which combined features from both segmented and original images to classify samples into malignant or benign categories along with specific ALL subtypes, including malignant‐pre‐B, malignant‐early pre‐B and malignant‐pro‐B. However, despite providing comprehensive analysis for ALL identification and subtype classification, the multi‐step deep learning architecture has several drawbacks, including high processing requirements and a reliance on the domain knowledge.

A multi‐step deep neural networks structure was developed in another study [19] to identify acute myeloid leukaemia (AML) and common mutation statuses in AML cases by segmenting leukaemia cells from bone marrow images. There were 236 healthy controls among the 1,487 newly identified AML cases in the dataset. In order to train a faster region‐based convolutional neural network (FRCNN) model, haematologists manually collected features from an upgraded dataset that included 5202 AML pictures and 5428 healthy samples. The binary classification model distinguished between bone marrow samples from AML and healthy ones with an accuracy of 91% and 86%, respectively, in predicting the NPM1 mutation status. Even with these encouraging outcomes, the manual feature extraction procedure was labourious and prone to mistakes, especially when working with big datasets.

Mondal et al. [20] investigated the use of ensemble methods in conjunction with deep learning to predict ALL and its subtypes using PBS imagery. To solve the class imbalance problem, an oversampling technique was implemented to the CNMC‐2019 dataset, yielding a training set of 11,644 photos. For transfer learning, the study used pre‐trained networks such as InceptionResNet‐V2, MobileNetV2, VGG‐16, Xception and DenseNet‐121. After integrating these networks, the ensemble model showed improved performance in detecting ALL, with an accuracy of 86.2% as well as an AROC value of 94.1%. The results showed that ensemble learning greatly increases the overall efficacy of models for predicting ALL using medical images by utilizing the advantages of various network designs.

An approach to pattern detection in ALL using computational deep learning was recently presented in another work [1]. The authors developed the ALLDM model, which analyses white blood cell count data to identify patterns indicative of ALL, such as changes in cell counts, composition, and protein or gene expression levels. The model achieved varying success rates across different treatment management strategies, with performance metrics ranging from 63.77% to 94.31% across two datasets. However, the study's reliance on white blood cell count data may limit its applicability, as it does not incorporate image‐based analyses, which are crucial for comprehensive ALL diagnosis. In our research, we aim to develop a model that surpasses the performance achieved by Jiwani et al. [1] by integrating image‐based data with advanced ensemble learning techniques, thereby enhancing diagnostic accuracy and robustness.

A comprehensive colour normalization technique for hematoxylin and eosin (H&E) stained histopathology pictures was presented by Vijh et al. [21] in 2021. Accurate image analysis can be hampered by colour differences caused by hand tissue sectioning and variable staining procedures, which their method overcomes. Three stages comprises this proposed approach: modified spectral normalization, fuzzy‐based stain normalization and enhanced fuzzy illuminant normalization. This approach efficiently reduces colour and stain variability, outperforming traditional normalization strategies, as shown by extensive simulations confirmed using histopathology images. However, the method's multi‐phase structure might add processing complexity, which might restrict its real‐time usefulness in clinical situations.

A method called spectral matching was created by Tosta et al. [22] in 2019 to fix the brightness of faded H&E‐stained histology pictures. The study discusses the difficulties caused by colour changes introduced by tissue preparation, digitalization and storage, which might have a negative impact on the processing of histological images. The suggested approach combines dictionary learning, fuzzy theory and the Cuckoo search algorithm to estimate the representation and concentration of H&E stain in image pixels. This method successfully restores coherent colour representation in faded samples without adding noise, according to visual and quantitative assessments. However, large‐scale implementations may need to take into account the substantial computational resources that the dependence on advanced optimization techniques may demand.

A comparative study of different colour normalizing techniques, including the Vahadane method, in the context of digital pathology whole‐slide images was carried out in 2020 by Ziaei et al. [23]. The goal of the study was to mitigate colour differences caused by various tissue types, staining techniques and scanning equipments. These variations can have an impact on pathologist interpretations as well as the robustness of AI algorithms. To align images from several scanners, the researchers utilized colour normalization techniques. The CIE colour difference (ΔE) metric was used to evaluate the normalization errors. The results showed that while more sophisticated approaches like StainGAN achieved more noticeable improvements, more conventional approaches like Vahadane only slightly lessened colour discrepancies. However, implementing such advanced methods may involve increased computational complexity and require substantial training data, which could be a limitation in certain scenarios, making Vahadane a great choice for our task.

A summary table including contribution and limitation is shown in Table 1.

**TABLE 1:** Recent research using machine learning and deep learning techniques for classifying acute lymphoblastic leukaemia.

Reference	Contribution	Limitations
Sampathila et al. (2022) [3]	Proposed ALLNET, a deep learning network for ALL detection trained on the ISBI 2019 dataset with 95.24% accuracy.	Did not classify leukaemia cells into specific subtypes.
Jha et al. (2019) [15]	Developed a hybrid leukaemia detection model using CNNs optimized by sine cosine algorithm (SCA), improving detection accuracy.	Required significant domain expertise and did not classify leukaemia into subtypes.
Atteia et al. (2022) [16]	Implemented Bayesian optimization to improve CNN hyperparameters, enhancing accuracy for ALL detection.	Did not extend scope to identify specific ALL subtypes.
Khandekar et al. (2021) [17]	Used YOLO v4 to detect ALL blast cells, achieving mean average precision (MAP) of 98.7% and 96.06% on different datasets.	Further improvements needed in diagnostic accuracy and robustness.
Ghaderzadeh et al. (2022) [18]	Developed a DenseNet‐201 model for ALL subtype classification using PBS images.	High processing requirements and reliance on domain knowledge.
Eckardt et al. (2022) [19]	Used faster R‐CNN to classify AML cases with 91% accuracy and predict NPM1 mutation status with 86%.	Manual feature extraction was labour‐intensive and prone to errors.
Mondal et al. (2021) [20]	Applied ensemble learning with transfer learning for ALL detection, achieving 89.72% accuracy.	Model performance depended on the selection of pre‐trained networks and the proposed structure is too much computationally expensive for real time applications.
Jiwani et al. (2023) [1]	Developed the ALLDM model to analyse WBC count data for ALL detection.	Did not incorporate image‐based analyses, limiting comprehensive diagnosis.
Vijh et al. (2021) [21]	Proposed a multi‐step colour normalization technique to enhance histopathology image analysis.	Increased processing complexity, limiting real‐time applicability.
Tosta et al. (2019) [22]	Introduced spectral matching for brightness correction in H&E‐stained images.	Required significant computational resources for large‐scale applications.
Ziaei et al. (2020) [23]	Compared different colour normalization techniques, highlighting Vahadane as a viable option.	Advanced methods like StainGAN required high computational power and large datasets.

## 3 Methodology

This section details the network architecture, image processing, and methodology for classifying ALL, B‐ALL and subtypes. Data augmentation enhances the dataset, followed by feature extraction using VGG16. Pre‐trained and modified classifiers then classify the samples, with a website developed for real‐time classification.

**FIGURE 1:**
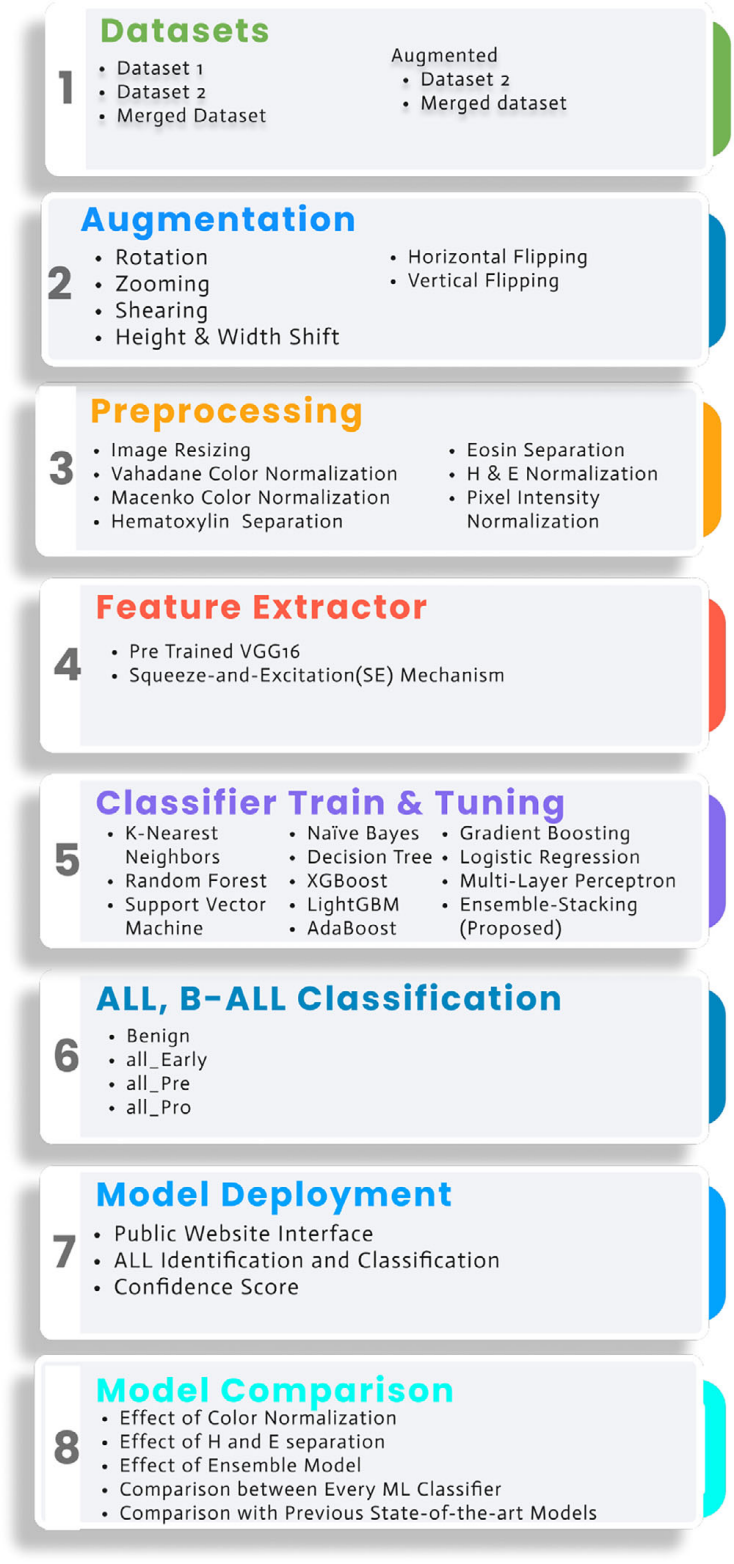
Methodology block diagram.

A summarized graphical representation of the methodology is provided in Figure 1 for clarity.

### 3.1 Data Collection

#### 3.1.1 Dataset 1

This dataset is collected from the study's PBS image dataset [24], which was sourced from Kaggle. The dataset consists of a total of 20,000 images, evenly distributed across four classes: Benign, All_Early, All_Pro and All_Pre. Each class contains 5000 images, ensuring a balanced representation for classification tasks.

#### 3.1.2 Dataset 2

**FIGURE 2:**
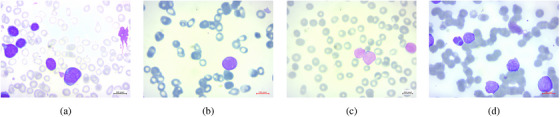
Randomly chosen from all categories of acute lymphoblastic leukaemia: (a) benign, (b) malignant early Pre‐B, (c) malignant Pre‐B and (d) malignant Pro‐B.

This dataset, collected from the study by Ghaderzadeh et al. [18], consists of 3256 images from 89 individuals suspected of having ALL, including 504 benign (haematogone) images and 2752 malignant images, with the malignant cases further distributed across Early Pre‐B (985), Pre‐B (963) and Pro‐B (804) subtypes. The bone marrow research laboratory [25] at Taleqani Hospital in Tehran, Iran, produced these pictures. Some randomly chosen sample images from each category are displayed in Figure 2.

#### 3.1.3 Merger Dataset

To create a more comprehensive dataset, dataset‐1, containing 20,000 images, was merged with dataset‐2, which contained 3256 images. This resulted in a combined dataset with a total of 23,256 images. The class distribution of the merged dataset is presented in Table 2.

**TABLE 2:** Class distribution in the merged dataset.

Class	Image count
Benign	5504
Early Pre‐B	5985
Pre‐B	5963
Pro‐B	5804
**Total**	**23256**

### 3.2 Data Preprocessing

In this study, the peripheral blood smear images were preprocessed by resizing, augmenting and normalizing to enhance the quality of the microscopic imagery data. The augmentation process was specifically applied to balance the dataset, ensuring unbiased results.

#### 3.2.1 Resizing

The input data are scaled to a predetermined size of 224 × 224 pixels before being analysed by our proposed model. This scaling stage guarantees consistency in input dimensions, which is necessary for effective processing and the network's best possible performance. The resolution of 224 × 224 pixels balances computational efficiency with the ability to capture important characteristics from every image [26].

#### 3.2.2 Handling Imbalanced Data Using Data Augmentation

In this study, the class imbalance problem was evident in one of the two datasets analysed. The first dataset “dataset 1”, consisting of 20,000 images evenly distributed across four classes (5000 images per class), did not require augmentation due to its balanced nature. However, the second dataset and merged dataset both exhibited a significant imbalance, with the early Pre‐B class having the highest number of samples and the benign class having the fewest.

To address this issue, various data augmentation techniques such as vertical and horizontal rotations, contrast and brightness adjustments, and random cropping, shearing and zooming were employed exclusively on the second dataset. Table 3 shows the data count before and after the augmentation process of dataset 2 and merged dataset.

**TABLE 3:** Image counts before and after augmentation for dataset‐2 and merged dataset.

	Dataset‐2		Merged dataset	
Class	Original count	After augmentation	Original count	After augmentation
Benign	504	2000	5504	6000
Early Pre‐B	985	2000	5985	6000
Pre‐B	963	2000	5963	6000
Pro‐B	804	2000	5804	6000
**Total**	**3256**	**8000**	**23,256**	**24,000**

To prevent data leakage, the dataset was first partitioned into training and test sets before any augmentation was applied. Augmentation techniques were strictly confined to the training partition only, ensuring that no augmented variants of training images were present in the test set. The test set retained only original, unmodified images throughout all experiments.

**FIGURE 3:**
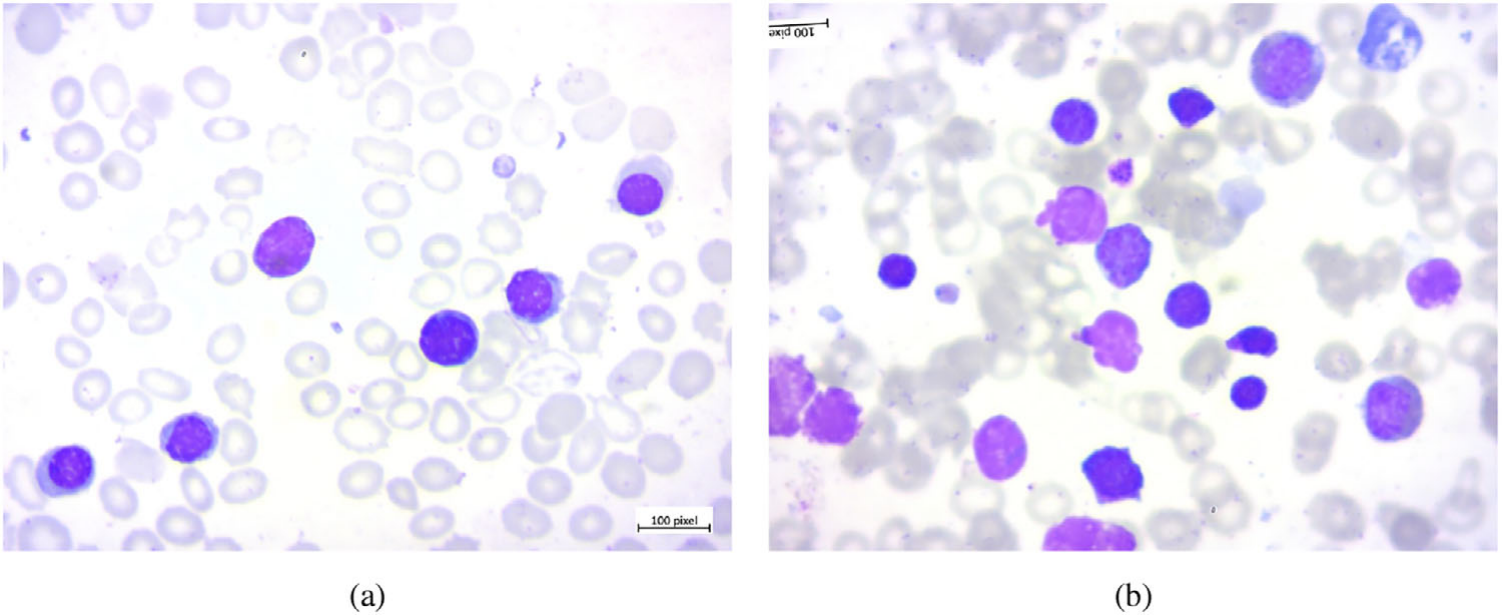
Sample data of (a) original data versus (b) augmented data.

By applying these methods, the dataset has been balanced across all classes along with increasing image count, enhancing the model's ability to learn representative features effectively from all categories. This approach not only mitigated the risks associated with biased predictions but also improved the model's overall performance and generalization capability in the presence of previously unseen data. Figure 3 shows an augmented image alongside the original image.

#### 3.2.3 Pixel Intensity and Stain Normalization Techniques

In this study, we applied multiple normalization techniques to address variability in histopathological image data, including pixel intensity scaling and advanced stain normalization methods. Each approach was systematically evaluated by replicating the dataset and applying distinct normalization strategies to identify optimal preprocessing for model performance.

##### 3.2.3.1 Basic Pixel Intensity Normalization

We have utilized the ImageDataGenerator function from Keras to rescale raw pixel values to the range [0, 1] by dividing each value by 255. This ensured uniformity in input magnitude and improved model convergence:

(1) $$I_{\text{norm}}=\frac{I_{\text{original}}}{255}$$

where $I_{\text{original}}$ represents raw pixel intensities.

##### 3.2.3.2 Stain‐Specific Normalization: H&E Separation

H&E stain separation was performed using an optical density (OD) transformation and singular value decomposition (SVD)‐based stain vector estimation [27]. The RGB image matrix $I\in R^{m\times n\times 3}$ was converted to OD space using the Beer–Lambert law:

(2) $$D=-\log_{10}\left(\frac{I+1}{I_{0}}\right),$$

where $I_{0}=240$ is the transmitted light intensity, and adding 1 prevents taking the logarithm of zero. Pixels with OD intensity less than a threshold β were removed:

(3) $$D_{\text{hat}}=\left\{D_{ij}|\min\left(D_{ij}\right)\geq \beta \right\}.$$

Next, SVD was applied to the covariance matrix of $D_{\text{hat}}$ to determine eigenvectors:

(4) $$USV^{T}=\text{SVD}\left(\text{Cov}\left(D_{\text{hat}}\right)\right).$$

The first two principal components were selected to define a projection plane. Data was projected onto this plane and normalized:

(5) $$T=D_{\text{hat}}V_{\left[:,1:2\right]}.$$

The angle of each point was computed with respect to the first SVD direction:

(6) $$\phi =\tan^{-1}\left(\frac{T_{\left[:,2\right]}}{T_{\left[:,1\right]}}\right).$$

The αth and (100−α)th percentiles of ϕ were used to estimate the stain vectors:

(7) $$v_{H},v_{E}=V_{\left[:,1:2\right]}\left[\begin{array}{cc} \cos(\phi_{\min}) & \cos(\phi_{\max})\\ \sin(\phi_{\min}) & \sin(\phi_{\max}) \end{array}\right].$$

Stain concentrations were computed using least squares estimation:

(8) $$C=V^{-1}D.$$

Finally, the stain concentrations were normalized using reference concentrations $C_{\text{ref}}$ and mapped back to OD space before reconstruction:

(9) $$D_{\text{norm}}=V_{\text{ref}}\left(C⊘C_{\text{ref}}\right).$$

The reconstructed image was obtained using the inverse Beer–Lambert law, ensuring robust stain separation and normalization across different images [27].

**FIGURE 4:**
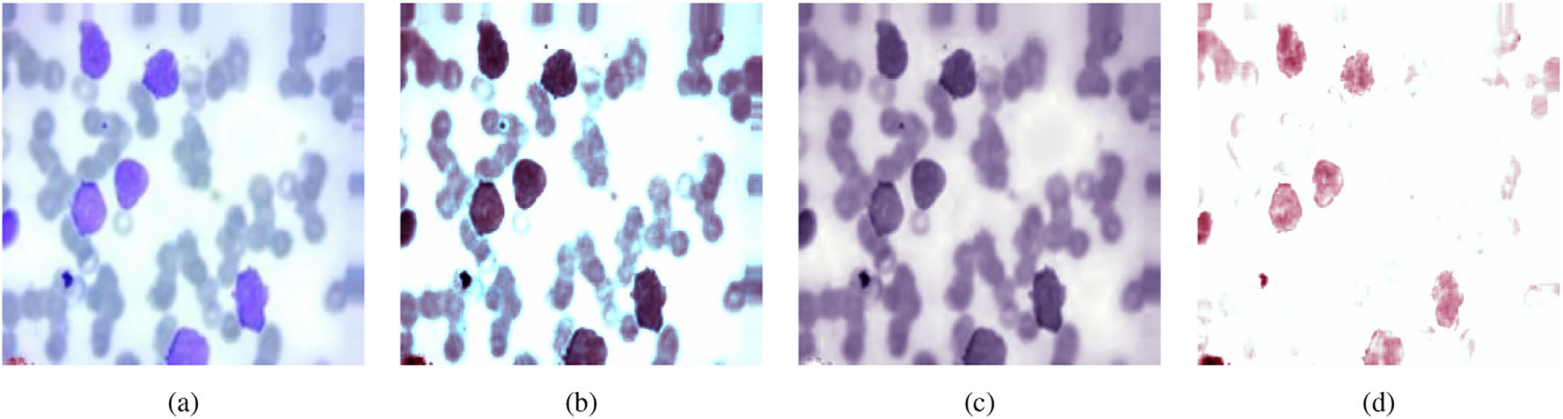
(a) Original PBS image, (b) H&E normalized image, (c) hematoxylin‐separated image, (d) eosin‐separated image.

The output of stain‐specific normalization, specifically H&E separation alongside with original image is shown in Figure 4.

##### 3.2.3.3 Macenko Stain Normalization

Macenko stain normalization utilizes the Beer–Lambert law to compute the optical density (OD) [28]:

(10) $$\text{OD}=-\log\left(\frac{I+1}{I_{0}+1}\right),$$

where I represents the observed intensity and $I_{0}$ denotes the background intensity. After filtering out low‐OD pixels, the covariance matrix C of the OD values is computed and decomposed using singular value decomposition (SVD):

(11) $$C=VS^{2}V^{T}.$$

The first two eigenvectors (columns of V) define the stain vectors, which are then used to normalize stain concentrations [28].

**FIGURE 5:**
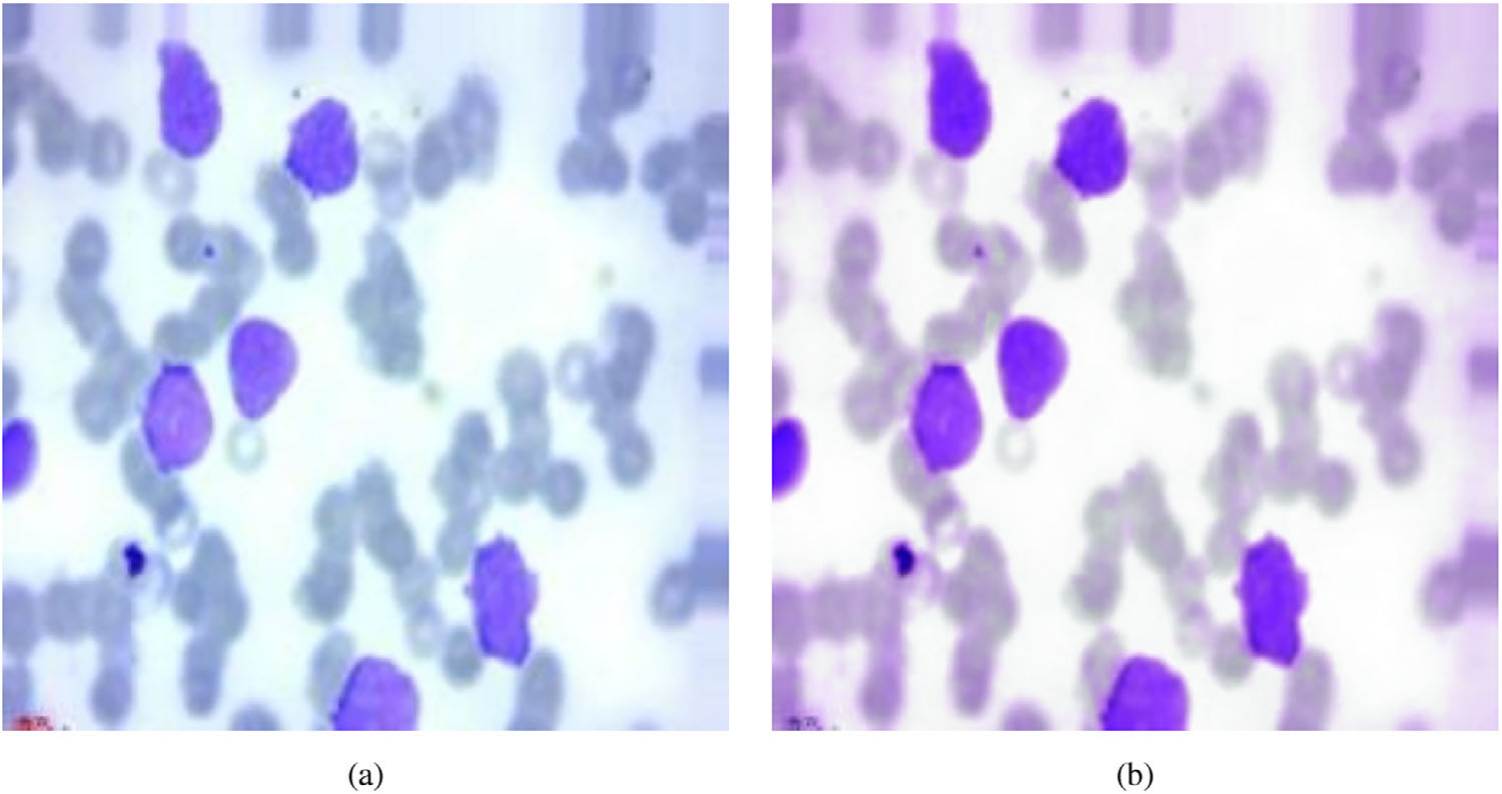
(a) Original PBS image, (b) colour normalized image using Macenko method.

In our work, we have employed the staintools library to implement Macenko normalization. A representative reference image was selected from our dataset to establish the target stain matrix. This matrix was then applied to normalize all other images, ensuring a uniform stain appearance across the dataset. This preprocessing step is crucial for reducing colour‐induced variability, thereby improving the robustness of subsequent image analysis tasks. The output of Macenko colour normalized image along side with original image is shown in Figure 5.

##### 3.2.3.4 Vahadane Stain Normalization

Vahadane et al. [29] proposed a stain normalization technique that decomposes the optical density (OD) matrix A into stain colour basis W and stain concentration matrix H using non‐negative matrix factorization (NMF):

(12) A=WH,W≥0,H≥0.

Here, W represents the stain colour basis, and H encodes the relative contribution of each stain in the image. The method involves the following steps:

1. Conversion to optical density space: The RGB image I is transformed to the OD space using:

(13) $$A=-\log\left(\frac{I}{I_{0}}\right)$$

where $I_{0}$ is the background illumination intensity.

2. Stain matrix estimation: The OD matrix A is factorized into W and H using NMF, ensuring non‐negativity constraints on both matrices.

3. Stain separation and normalization: The estimated stain matrix W from a reference image is used to transform new images, aligning their stain appearances to the reference while preserving morphological details.

**FIGURE 6:**
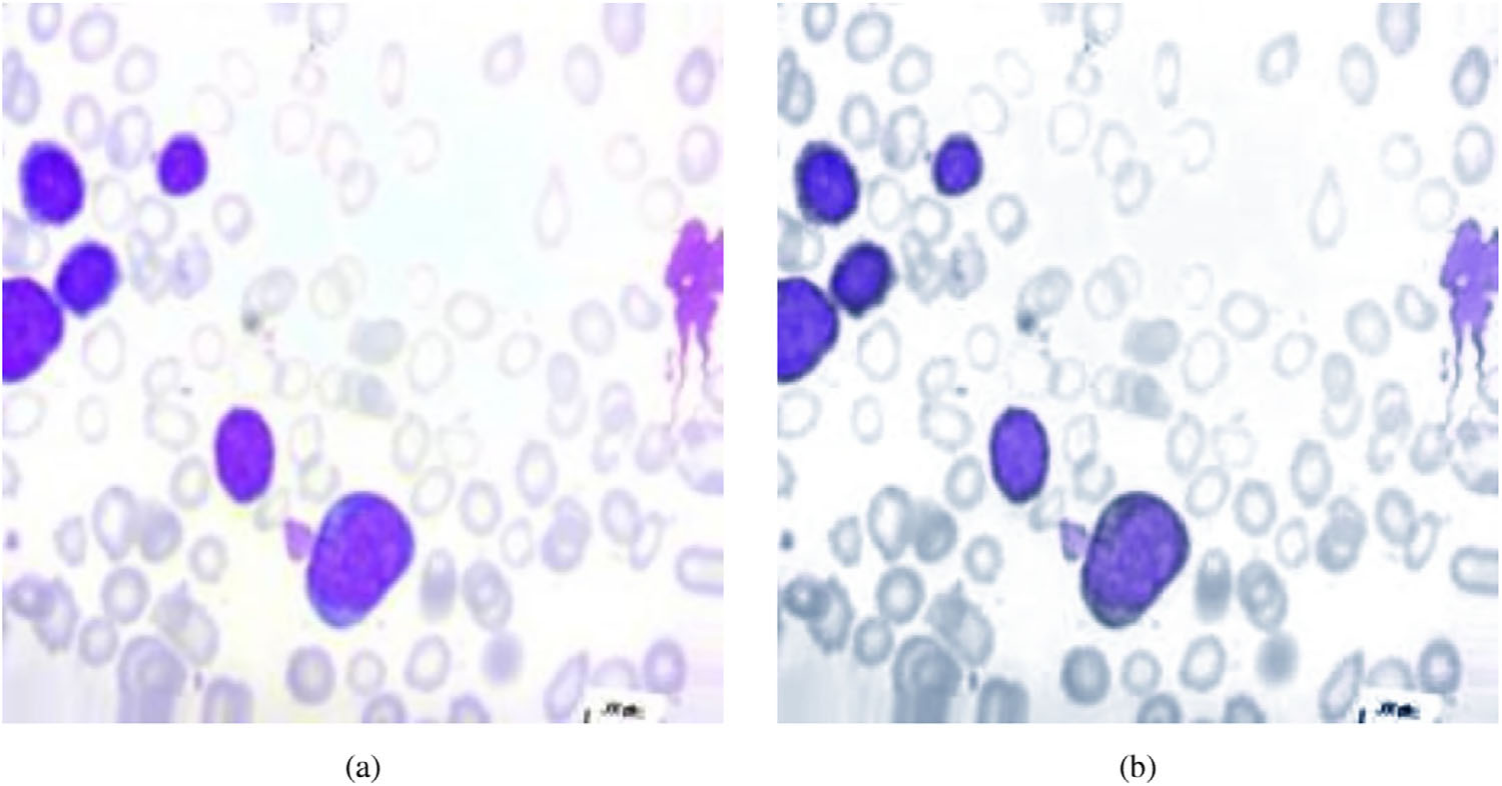
(a) Original PBS image, (b) colour normalized image using the Vahadane method.

In this work, the Vahadane method is implemented in a manner similar to Macenko normalization using the staintools library. A reference image was chosen to determine the target stain matrix, which was then applied to standardize the appearance of all images in the dataset. This approach helped mitigate staining variability, enhancing consistency across samples. The output of Vahadane colour normalized image alongside with original image is shown in Figure 6.

The original dataset is replicated five times, with each copy processed using one normalization technique (H separation, E separation, H&E normalization, Macenko and Vahadane). Pixel scaling is employed in every dataset. This enabled direct comparison of model performance (accuracy, generalizability) across preprocessing strategies. All methods were applied prior to data augmentation to ensure consistency in downstream transformations.

### 3.3 Feature Extraction

A pre‐trained model known as the visual geometry group 16 (VGG16) is employed as a feature extractor. The visual geometry group at the University of Oxford first presented this convolutional neural network (CNN) architecture for image categorization [30]. Thirteen convolutional, three fully connected and 16 weight layers make up the deep structure of this model. The architecture is organized into blocks of convolutional layers with small receptive fields (3×3), interspersed with max‐pooling layers. The model can learn intricate, hierarchical features from input data to its deep design.

Each block of convolutional layers is followed by max‐pooling layers, effectively decreasing the feature maps' spatial dimensions, which enhances computational efficiency and introduces translation invariance. The final layer of the architecture is usually the output layer for classification tasks, where fully linked layers process the gathered features to produce predictions. The network makes use of the rectified linear unit (ReLU) activation function, which adds non‐linearity and facilitates the discovery of complex data correlations.

**FIGURE 7:**
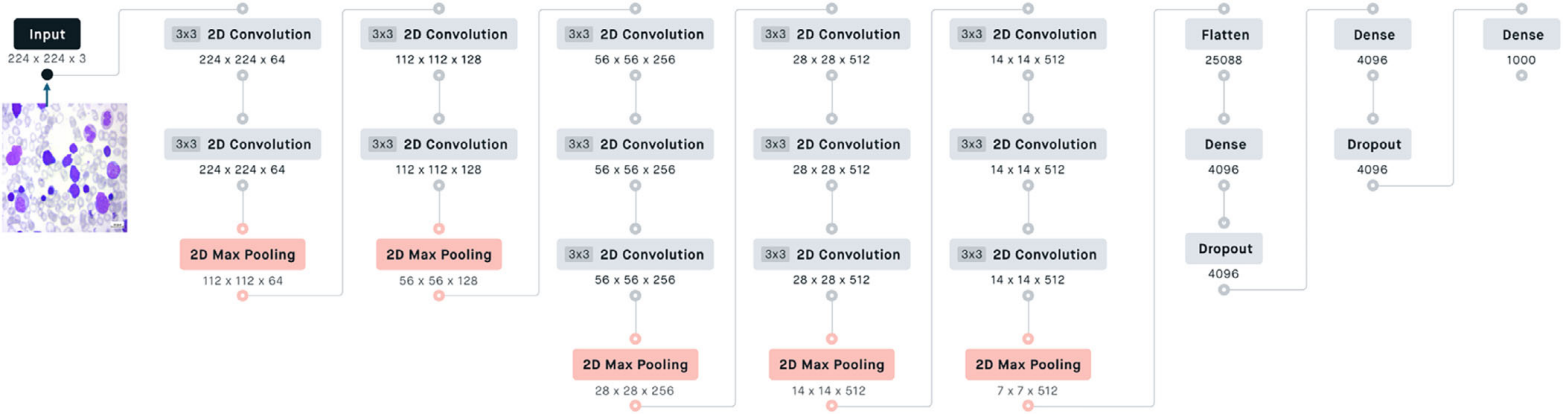
VGG16 architecture.

VGG16 has proven highly effective at extracting and interpreting detailed hierarchical features from image type data, making it extremely well‐suited for detection and classification tasks, such as skin cancer or blood cancer diagnosis. Compared to traditional methods like the ABCDE criteria [31], the “ugly duckling” rule [32], 3D textured mesh analysis [33] and biopsies [34], VGG16 demonstrates superior capability in identifying critical patterns indicative of skin lesions. Its hierarchical convolutional layer structure makes it possible to automatically extract relevant information, which increases its usefulness in applications that analyse intricate medical images. The VGG16 architecture is shown in Figure 7.

The model was imported with weights gained from the ImageNet dataset, removing the top fully connected layers (include_top = False), in order to adapt the VGG16 architecture for the particular classification task. The input shape was adjusted to (224, 224, 3), suitable for the dataset's image dimensions. The model was not retrained; it was solely utilized for feature extraction using pre‐trained weights, thus requiring no additional training time. This configuration allows the model to use pre‐trained feature extraction capabilities while adapting to the dataset's unique requirements.

**FIGURE 8:**
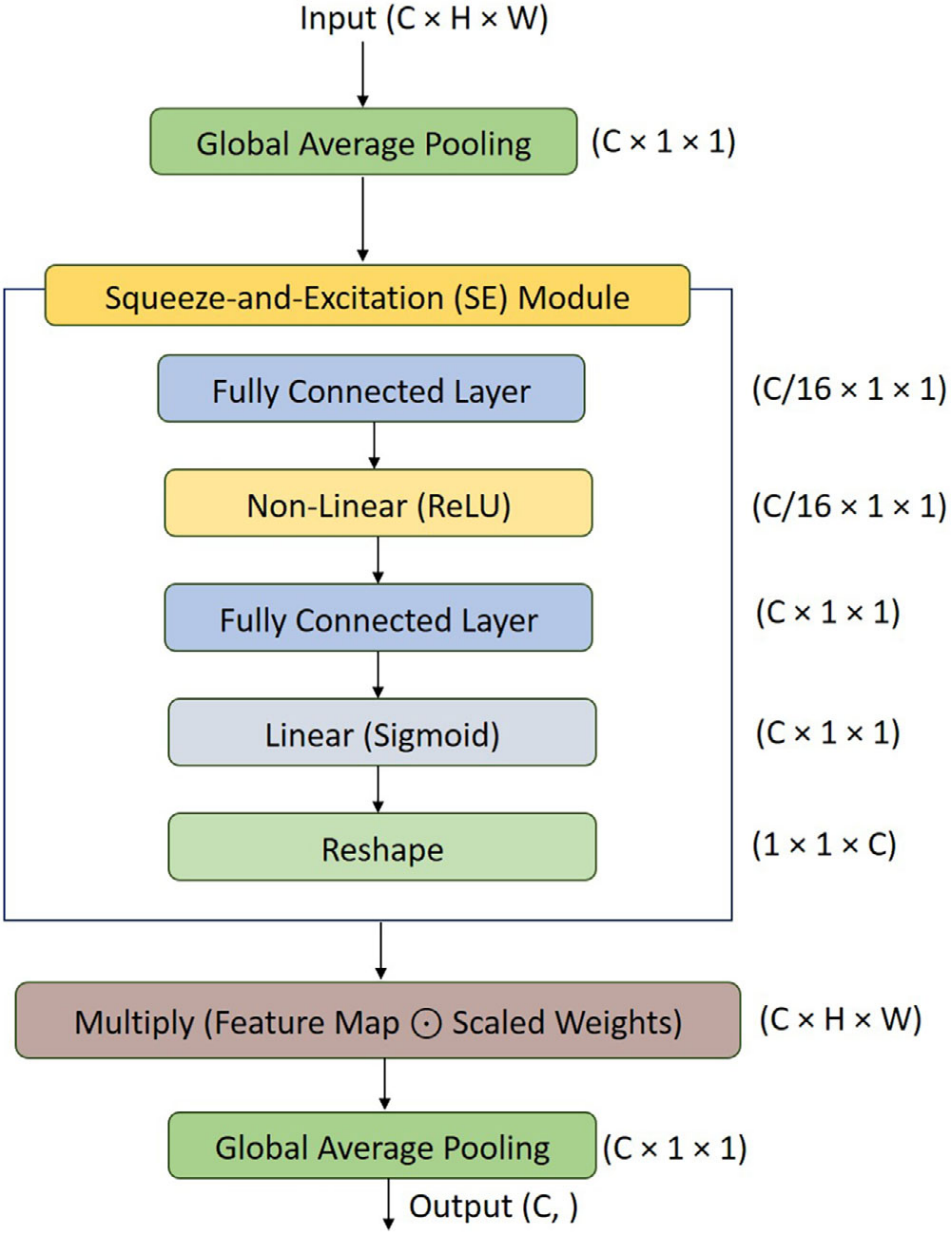
Squeeze‐and‐excitation (SE) mechanism.

To further refine feature extraction and improve compatibility with machine learning classifiers, additional layers were integrated. A global average pooling (GAP) layer was added to reduce spatial dimensions, followed by a squeeze‐and‐excitation (SE) mechanism to emphasize significant feature channels shown in Figure 8.

The recalibrated features were then processed through another GAP layer to produce one‐dimensional outputs optimized for classification by machine learning algorithms. These enhancements improve the feature extraction ability of the model for the task.

### 3.4 Classifier

#### 3.4.1 K‐Nearest Neighbours

In K‐nearest neighbours (KNN), classification decisions are made based on the distance between data points, with similar or identical samples having a smaller distance compared to dissimilar ones [35, 36]. KNN typically assigns the appropriate class label after determining the closest neighbours using Euclidean distance. Although KNN is simple to use, due to the must compute the distances to every data point, its effectiveness declines when dealing with big datasets. Furthermore, KNN's performance may be impacted by its sensitivity to noise or irrelevant features [37, 38]. Studies [39, 40] show how KNN may be used to effectively classify acute lymphoblastic leukaemia (ALL). In this research, KNN is used with a K value as 10. The choice of K=10 was determined through empirical evaluation, where it provided a balance between bias and variance. A larger K value helps reduce the impact of noise and outliers, leading to more stable and robust classification results, while still maintaining sufficient sensitivity to local variations in the data.

#### 3.4.2 Random Forest

A popular and reliable machine learning technique, random forest (RF) is renowned for its high efficacy and accuracy [41]. It is a tree‐based classifier in which a random subset of the features and data is used to build each unique tree. By combining the predictions from every tree in the forest, the final classification choice is made, enhancing overall performance and minimizing overfitting. Random forest was used to distinguish between normal and malignant lymphoblasts in a study [42]. Additionally, to further increase the prediction accuracy for the categorization of B‐acute lymphoblastic leukaemia (ALL), the Adaboost‐enhanced random forest (ADBRF) model has been implemented [43]. In this study, the random forest classifier has been utilized with 100 estimators to classify the features extracted from the VGG16 model. The classifier's behaviour was controlled using a fixed random state 42, ensuring reproducibility of results.

#### 3.4.3 Support Vector Machines (SVM)

SVM, an increasingly common supervised learning approach, which is utilized for classification problems [44, 45]. Hyperplanes are used to divide classes, with the goal of maximizing the margin among feature vectors from various classes and the hyperplane. This is achieved by ensuring that the separation satisfies the condition‐

(14) $$y_{i}\left(w\cdot x_{i}+b\right)>1$$

where $y_{i}$ denotes the associated class label and $x_{i}$ is the input feature vector. When this condition is equal to 1, the support vector is identified. When the data is linearly separable, SVM performs well; in non‐linear circumstances, it maps the data into a higher‐dimensional space using kernel functions, which improves classification. The choice of kernel and its parameters are crucial for achieving optimal results, with radial basis function (RBF) kernels often employed for this purpose [35, 44]. SVM has been effectively applied in classifying ALL into its subtypes, as demonstrated in a study [46], where they used ensemble SVM classifiers to classify ALL subtypes successfully. In this study, a linear kernel was used in the SVM model with the probability option enabled, allowing for better classification performance.

#### 3.4.4 Gradient Boosting Classifier

Gradient boosting is an ensemble strategy that improves the accuracy and robustness of weak models, usually decision trees, by successively combining them to generate a strong prediction model [47]. In each iteration, the model focuses on correcting the errors made by the previous model, effectively improving its predictive accuracy. Depending on the particular requirements of the application, this approach can incorporate different base learners, making it very successful for a variety of machine learning applications [47]. In this study, gradient boosting was implemented with 100 estimators and a fixed random state of 42, ensuring consistency in the results across different runs.

#### 3.4.5 Logistic Regression

A robust statistical technique called logistic regression models the connection between predictor variables and a binary result using a sigmoid function [48]. This method transfers input information to a probability value between 0 and 1, making it especially appropriate for binary classification tasks. The Equation (15) demonstrates how the relationship is expressed mathematically,

(15) $$P\left(y=1|x\right)=\frac{1}{1+e^{-(w^{T}x+b)}}$$

where the weight vector is denoted by w, the input feature vector by x, the bias term by b, and the probability of the positive class by P(y=1∣x).

The model's coefficients (w and b) represent how changes in the input variables affect the log‐odds of the dependent variable, providing insight into the influence of each predictor on the final probability. In this study, the logistic regression model was used with a maximum of 1000 iterations and a fixed random state of 42, ensuring consistent results across different model runs. This model is effective in classifying data by transforming features into probabilistic outputs [48], making it a widely adopted approach in various machine learning applications.

#### 3.4.6 Decision Tree Classifier

Decision tree (DT) is a prominent classification system that arranges data in a tree structure. In DT the internal nodes represents the features and leaf nodes represents the labels of the data samples. It divides difficult decisions into easier ones using a divide‐and‐conquer strategy [35, 36]. The root node, which concentrates on a particular pattern property, is where the categorization process begins. The subsequent decisions are made at descendant nodes based on different possible values, continuing until a leaf node is reached. One of the most common algorithms used for decision tree classification is C4.5, and its variant EC4.5 is considered more robust, with a faster training phase than neural networks [49]. However, DT models can be sensitive to noise, and their performance can be unstable with small data variations. In this study, a decision tree classifier with a maximum depth of 10 was implemented for efficient classification of ALL, specifically B‐ALL.

#### 3.4.7 Naïve Bayes

Based on statistical principles, naïve Bayes (NB) is a common supervised classification method, where the relationships between variables are expressed through probabilities rather than direct predictions [35, 36, 50]. When using naïve Bayes, features are depicted as nodes in a graph, while the relationships among them are shown as arcs, and typically, each feature is assumed to be conditionally independent of others, given a single parent node. This method is computationally efficient, both during training and testing, and is particularly robust in handling missing values, as it disregards them during probability estimation, ensuring that the final decision remains unaffected by incomplete data [36]. In the context of B‐acute leukaemia classification, naïve Bayes has been successfully applied to categorize various leukaemia subtypes and healthy white blood cell types, demonstrating its utility in medical diagnostics. In this study, a Gaussian naïve Bayes classifier is employed for ALL classification tasks.

#### 3.4.8 Multi‐Layer Perceptron (MLP)

One kind of feed‐forward artificial neural network (ANN) is the MLP, which is made up of several layers of connected neurons, each of which has an activation function that is based on a threshold. A minimum of three layers are usually present in an MLP: an input layer, one or more hidden levels, and an output layer. In contrast to linear perceptrons, MLP implement non‐linear activation functions to identify intricate correlations in the data [51]. The network is trained using supervised learning techniques, particularly backpropagation, to adjust the weights and biases for accurate predictions. Each neuron in the network receives inputs, applies a weight, adds a bias and passes the result through an activation function (e.g. sigmoid function) to produce the output [51, 52]. The mathematical representation of an MLP is expressed as:

(16) $$y=\phi \left(\sum_{i=1}^{m}w_{i}x_{i}+b\right)$$

where b is the bias term, $w_{i}$ are the associated weights, and $x_{i}$ stands for the input values. In this study, an MLP classifier with a hidden layer configuration of (128, 64) and a maximum of 1000 iterations was used to classify the data effectively. With this configuration, the model can identify complex patterns in the data and generate accurate classification.

#### 3.4.9 Extreme Gradient Boosting

The ensemble learning algorithm extreme gradient boosting (XGBoost) is scalable and extremely effective [53]. It is currently among the most used machine learning methods, especially in competitive data science environments like Kaggle. XGBoost is designed to optimize the gradient boosting framework by introducing regularization into the objective function, improving model performance and reducing overfitting. This method has been successfully applied to both regression and classification tasks, delivering state‐of‐the‐art performance in various machine learning competitions [53]. For this study, the XGBClassifier was employed with 100 estimators, a learning rate of 0.1, and a random state of 42 to ensure reproducibility and robust performance.

#### 3.4.10 Light Gradient Boosting Machine (LGBM)

LGBM is an efficient and high‐performance gradient boosting framework that is optimized for speed and memory usage [54]. It is particularly well‐suited for large datasets and resource‐constrained environments due to its ability to process data quickly with minimal memory consumption. One of the main characteristics of LGBM is that it bins continuous feature values using a histogram‐based method, reducing the computational cost and memory footprint while maintaining high accuracy. This makes LGBM an ideal choice for situations where both time efficiency and scalability are important. In this study, the LGBM classifier was utilized with 100 estimators, a learning rate of 0.1 and a random state of 42 parameter.

#### 3.4.11 Proposed Stacking Model

**FIGURE 9:**
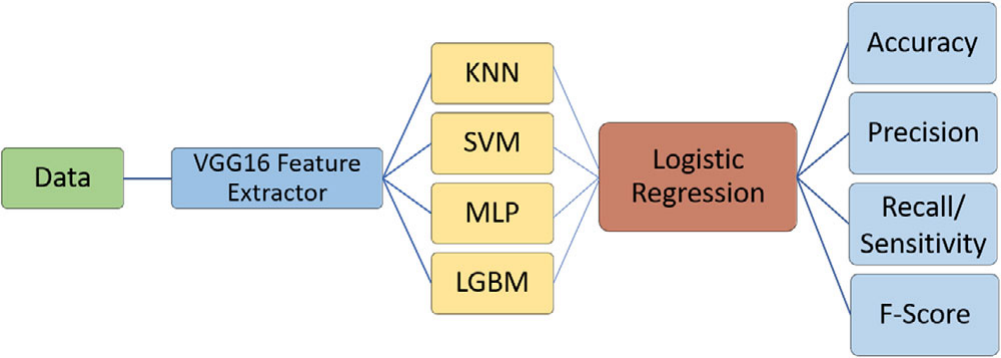
Stacking classifier model's stage.

An ensemble classifier boosts classification performance or accuracy by combining the strengths of various models or incorporating multiple kernel functions within a unified system. For instance, Adaboost combines weak classifiers to create a stronger model, reducing the overall error rate [43]. In this study, an ensemble model is constructed using four top‐performing classifiers based on accuracy: K‐nearest neighbour, support vector machine, multilayer perceptron, and LightGBM. The final prediction is made by a logistic regression model, which acts as the meta‐classifier, ensuring a more robust classification output. This stacking approach optimizes the performance by combining the diverse strengths of these individual models. The structure of the model is shown in Figure 9.

### 3.5 Performance Evaluation Matrix

The effectiveness of the proposed method was assessed using the following performance metrics:

#### 3.5.1 Confusion Matrix

A confusion matrix is a structured table that compares expected and actual results to assess how well categorization models perform. It is commonly used to evaluate classification models.

##### 3.5.1.1 Accuracy

Accuracy evaluates the classifier's effectiveness in correctly predicting class labels. It is computed using the formula stated in Equation (17)

(17) $$\text{Accuracy}=\frac{\text{TN}+\text{TP}}{\text{FN}+\text{FP}+\text{TN}+\text{TP}}$$

##### 3.5.1.2 Precision

Precision is a performance statistic that assesses how well a classifier makes positive predictions. The ratio of actual positive predictions to all predicted positives is how it is defined. When the classifier predicts the positive class with high precision, it makes fewer false positive errors. Equation (18) illustrates how to compute precision.

(18) $$\text{Precision}=\frac{\text{TP}}{\text{FP}+\text{TP}}$$

##### 3.5.1.3 Sensitivity or Recall

Sensitivity is widely used in metrics in medical and epidemiological research, although they are less recognized in some mathematical fields. Sensitivity assesses the classifier's ability to identify the positive class. It is calculated using the formula stated in equation 19.

(19) $$\text{Sensitivity or recall}=\frac{\text{TP}}{\text{FN}+\text{TP}}$$

##### 3.5.1.4 F1‐Score

The F1‐score is a statistical measure that is used to evaluate prediction accuracy by combining recall and precision. A balanced performance metric, the F1‐score is calculated as the harmonic mean of recall and precision. In Equation (20), the formula for the F‐score is shown.

(20) $$F1-\text{Score}=2\times \frac{\text{Precision}\times \text{Recall}}{\text{Precision}+\text{Recall}}$$

### 3.6 Explainable AI (LIME and Grad‐CAM++)

#### 3.6.1 LIME

LIME is a technique designed to provide interpretable insights into the decision‐making processes of complex machine learning models. It works by perturbing the input image through binary variations, generating a range of predictions from the model. A simplified linear regression model, weighted by cosine distances, approximates the predictions of the complex model. By identifying and visualizing key super‐pixels, LIME highlights the regions that significantly influence the model's decisions. The LIME optimization process mathematically described by the following equation:

(21) $$\hat{g}\left(x^{\prime }\right)=\arg\min_{g\in G}\left[L\left(f,g,\pi_{x}\right)+\Omega \left(g\right)\right]$$

where: f is the complex model, g is the interpretable model, L is the loss function that measures how well the interpretable model g approximates the predictions of the complex model f, $\pi_{x}$ is the neighbourhood around the instance x, Ω(g) is a complexity penalty on the model g.

#### 3.6.2 Grad‐CAM++

We utilized an enhanced version of Grad‐CAM known as Grad‐CAM++ in this study. This approach considers only positive gradients when calculating importance weights for feature maps. As a result, Grad‐CAM++ is better equipped to handle images with multiple instances of the same class, producing more accurate and detailed heat maps that indicate the critical areas influencing classification decisions. Additionally, Grad‐CAM++ modifies the original Grad‐CAM equation by incorporating refinement terms. The weights are calculated using the following equation:

(22) $$\alpha_{k}^{c}=\frac{1}{Z}\sum_{i}\sum_{j}\frac{\partial^{2}Y^{c}}{\partial A_{k}^{2}}$$

### 3.7 Computational Environment and Methodology Documentation

This section details the computing environment, libraries and parameters used during the research. Documenting technical details allows future researchers to confidently replicate and expand on our findings.

The following Tables 4 and 5 outlines the exact computational tools and configurations used in our analysis. Besides, Table 6 outline the hyper‐parameters utilized for each model.

**TABLE 4:** System configuration.

Component	Details
Processor	Intel Core i7‐6500U
RAM	8 GB
ROM	120 GB
Operating system	Windows 11 Pro 64‐bit

**TABLE 5:** Python libraries and versions.

**Package name**	**Version**	**Description**
Python	3.10.16	Programming language
matplotlib	3.10.0	Plotting and visualization
seaborn	0.13.2	Plotting and visualization
numpy	1.26.4	Numerical computations
scipy	1.15.1	Scientific computations
scikit‐learn	1.2.2	Machine learning library
tensorflow	2.17.1	Deep learning library (Keras)
py‐xgboost	2.1.3	XGBoost
staintools	2.1.2	Image staining and analysis

**TABLE 6:** Hyperparameters used during grid search for each model.

Model	Hyperparameter ranges
K‐nearest neighbours (KNN)	n_neighbours∈{3,5,10,15}
Random forest	n_estimators∈{50,100,200}, max_depth∈{None,10,20,30}
Support vector machine (SVM)	kernel∈{linear, rbf, poly}, C∈{0.1,1,10}
Gradient boosting (GB)	n_estimators∈{50,100,200}, learning_rate∈{0.01,0.1,0.2}
Logistic regression	C∈{0.1,1,10}, max_iter=1000
Decision tree	max_depth∈{None,5,10,20}, criterion∈{gini, entropy}
Naïve Bayes (NB)	Default hyperparameters
Multi‐layer perceptron (MLP)	hidden_layer_sizes∈{(64,),(128,64)}, max_iter=1000
XGBoost (XGB)	n_estimators∈{50,100,200}, learning_rate∈{0.01,0.1,0.2}
LightGBM (LGBM)	n_estimators∈{50,100,200}, learning_rate∈{0.01,0.1,0.2}
Extra trees (ET)	n_estimators∈{50,100,200}, max_depth∈{none,10,20,30}
AdaBoost	n_estimators∈{50,100,200}, learning_rate∈{0.01,0.1,1}
Stacking classifier	Base estimators: KNN, SVM, MLP, LGBM; final estimator: logistic regression

### 3.8 Medical Application and Real‐Time Classification

The integration of machine learning and deep learning techniques for diagnosing ALL, B‐ALL and its subtypes has the potential to revolutionize medical diagnostics, particularly when coupled with real‐time classification systems. Traditional diagnostic methods rely heavily on expert pathologists, which can be time‐consuming and prone to human error. The use of computational models enables automated, accurate and efficient classification from peripheral blood smear images, significantly aiding early classification and timely treatment.

**FIGURE 10:**
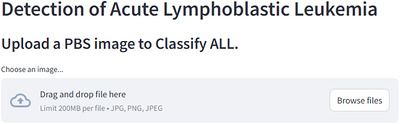
Proposed medical application for medical professionals.

To facilitate real‐time classification and accessibility, we have developed an interactive web‐based application using Streamlit, a lightweight and efficient framework for deploying machine learning models. The prototype application is accessible at https://allbcellclassify.streamlit.app. This application allows medical practitioners and researchers to upload blood smear images and obtain immediate predictions regarding the presence of ALL, B‐ALL and its subtype. Our proposed system not only classifies the input image but also provides a confidence score, indicating the model's certainty regarding the prediction and the intuitive interface enables seamless interaction for medical professionals with minimal technical expertise, ensuring ease of use in clinical settings. The default user interface of our proposed Medical Application is shown in Figure 10.

**FIGURE 11:**
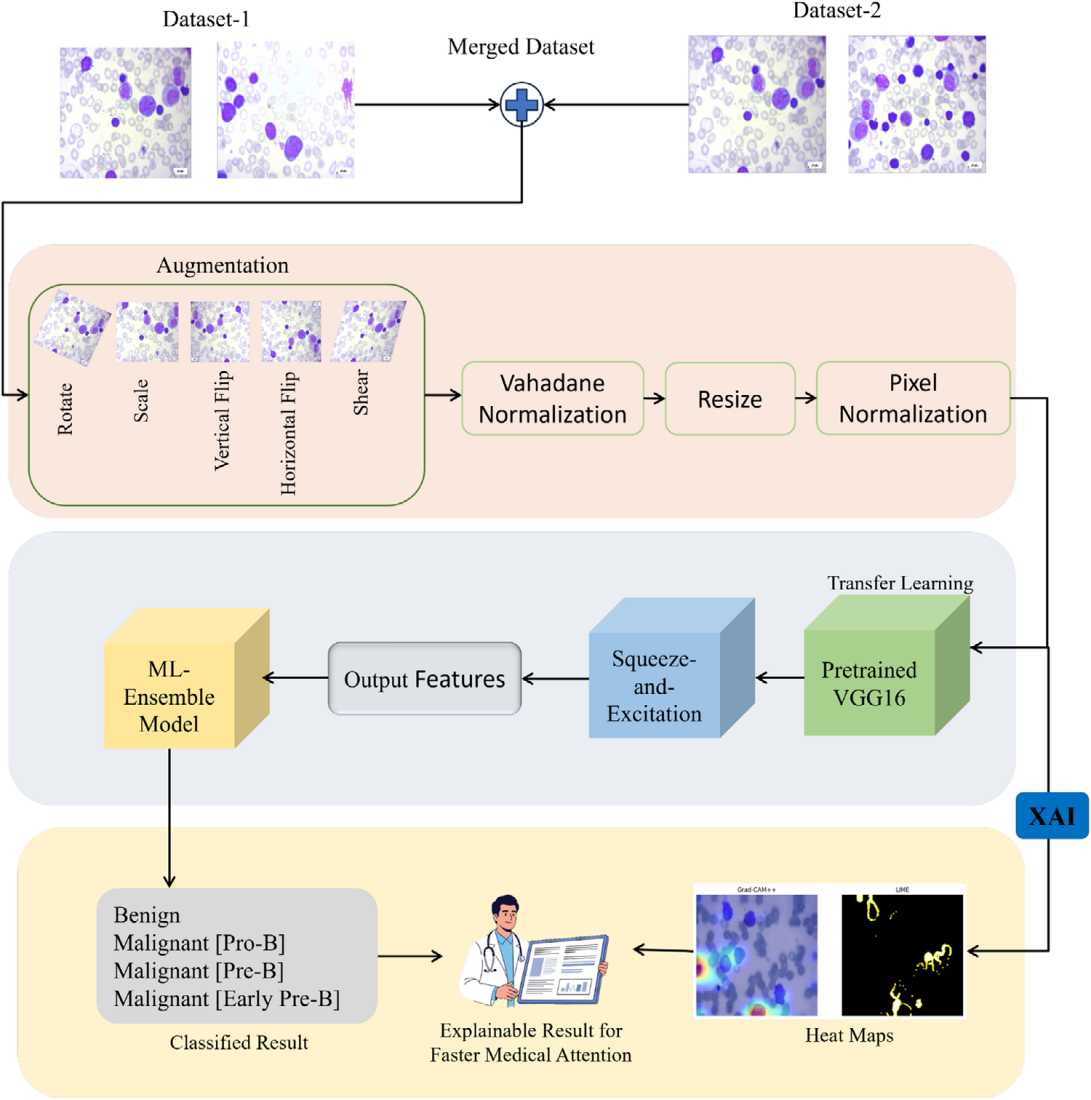
Overview of the proposed final model pipeline, including dataset merging, augmentation, stain normalization, preprocessing, transfer learning with VGG16, squeeze‐and‐excitation feature refinement, ML‐ensemble classification and XAI‐based interpretability.

Finally, the complete pipeline of the proposed framework is summarized in Figure 11, providing an end‐to‐end overview of data preprocessing, feature extraction, classification and explainability components. To further ensure reproducibility and transparent documentation of the exact order of operations followed throughout this study, the pipeline is also formalized in Algorithm 1.

ALGORITHM 1 **Complete methodology pipeline.**

**Table:** 

**Require**: Raw datasets $D_{1}$, $D_{2}$	
1:	Merge $D_{1}$ and $D_{2}$ to form $D_{\text{merged}}$
2:	Split each dataset into $D_{\text{train}}$ (80%) and $D_{\text{test}}$ (20%)
3:	Apply augmentation only to $D_{\text{train}}$ → $D_{\text{train}}^{\text{aug}}$
4:	Apply stain normalization (Vahadane/Macenko/H&E) to $D_{\text{train}}^{\text{aug}}$ and $D_{\text{test}}$ separately
5:	Extract VGG16 + SE block features from normalized $D_{\text{train}}^{\text{aug}}$ and $D_{\text{test}}$
6:	Train ML classifiers (KNN, SVM, MLP, LGBM, etc.) on $D_{\text{train}}^{\text{aug}}$ features
7:	Train stacking ensemble on $D_{\text{train}}^{\text{aug}}$ features
8:	Evaluate all classifiers on $D_{\text{test}}$ features
9:	Apply Grad‐CAM++ and LIME for explainability

## 4 Result and Discussion

The proposed methodology demonstrates exceptional performance in classifying ALL subtypes using a combination of machine learning classifiers for classification, and deep learning models for feature extraction from normalized PBS images. Among these classifiers, an ensemble model built using the top four best‐performing classifiers emerged as the most effective.

### 4.1 Performance Metrics

The 13 machine learning algorithms exhibited high performance, with accuracy ranging from 76.35% to 99.95%. Out of 13 machine learning algorithms, Stacking achieved the highest accuracy of 99.95%, 99.85%, 98.25%, 98.10% and 97.10% using the normalization techniques Vahadane, Macenko, H & E normalization, hematoxylin separation, and eosin separation sequentially for the balanced dataset 1. Besides, Out of 13 machine learning algorithms, again Stacking achieved the highest accuracy of 99.25%, 99.13%, 96.00%, 99.13%, and 93.63% using the normalization techniques Vahadane, Macenko, H & E normalization, hematoxylin separation, and eosin separation sequentially for the balanced dataset 2. Finally, Out of 13 machine learning algorithms again Stacking achieved the highest accuracy of 99.33%, 99.58%, 97.78%, 98.29% and 95.96% using the normalization techniques Vahadane, Macenko, H & E normalization, hematoxylin separation, and eosin separation sequentially for the balanced merged dataset. A comprehensive analysis of the results obtained from the testing phase is shown in Tables 7, 8, 9 for dataset 1, dataset 2 and merged dataset, respectively.

**TABLE 7:** Complete performance comparison of all models across different normalization applied for dataset‐1.

**Model**	**Normalization method**	**Accuracy**	**Precision**	**Recall**	**F1 score**
Stacking model	H separated	98.10%	98.11%	98.10%	98.10%
	E separated	97.10%	97.11%	97.10%	97.10%
	H and E normalized	98.25%	98.26%	98.25%	98.25%
	Macenko colour normalized	99.85%	99.85%	99.85%	99.85%
	Vahadane colour normalized	99.95%	99.95%	99.95%	99.95%
MLP	H separated	97.35%	97.37%	97.35%	97.35%
	E separated	95.70%	95.72%	95.70%	95.71%
	H and E normalized	97.70%	97.72%	97.70%	97.70%
	Macenko colour normalized	99.75%	99.75%	99.75%	99.75%
	Vahadane colour normalized	99.90%	99.90%	99.90%	99.90%
LGBM	H separated	97.25%	97.27%	97.25%	97.25%
	E separated	95.00%	95.03%	95.00%	95.01%
	H and E normalized	97.45%	97.45%	97.45%	97.45%
	Macenko colour normalized	99.50%	99.50%	99.50%	99.50%
	Vahadane colour normalized	99.70%	99.70%	99.70%	99.70%
XGB	H separated	96.60%	96.63%	96.60%	96.61%
	E separated	94.30%	94.32%	94.30%	94.31%
	H and E normalized	96.65%	96.65%	96.65%	96.65%
	Macenko colour normalized	99.15%	99.15%	99.15%	99.15%
	Vahadane colour normalized	99.45%	99.45%	99.45%	99.45%
KNN	H separated	95.95%	96.01%	95.95%	95.96%
	E separated	94.15%	94.15%	94.15%	94.14%
	H and E normalized	96.75%	96.76%	96.75%	96.75%
	Macenko colour normalized	99.20%	99.20%	99.20%	99.20%
	Vahadane colour normalized	99.75%	99.75%	99.75%	99.75%
SVM	H separated	94.95%	94.98%	94.95%	94.96%
	E separated	91.75%	91.79%	91.75%	91.74%
	H and E normalized	96.00%	96.01%	96.00%	96.00%
	Macenko colour normalized	98.75%	98.76%	98.75%	98.75%
	Vahadane colour normalized	99.30%	99.30%	99.30%	99.30%
Extra trees	H separated	93.90%	94.01%	93.90%	93.92%
	E separated	92.10%	92.12%	92.10%	92.09%
	H and E normalized	94.95%	94.99%	94.95%	94.94%
	Macenko colour normalized	97.95%	97.97%	97.95%	97.95%
	Vahadane colour normalized	99.25%	99.25%	99.25%	99.25%
Random forest	H separated	93.80%	93.98%	93.80%	93.83%
	E separated	92.05%	92.05%	92.05%	92.04%
	H and E normalized	94.75%	94.80%	94.75%	94.75%
	Macenko colour normalized	98.05%	98.06%	98.05%	98.05%
	Vahadane colour normalized	99.00%	99.00%	99.00%	99.00%
Gradient boosting	H separated	93.60%	93.63%	93.60%	93.60%
	E separated	91.65%	91.68%	91.65%	91.66%
	H and E normalized	95.05%	95.04%	95.05%	95.04%
	Macenko colour normalized	98.75%	98.76%	98.75%	98.75%
	Vahadane colour normalized	99.15%	99.15%	99.15%	99.15%
Logistic regression	H separated	93.10%	93.13%	93.10%	93.10%
	E separated	90.50%	90.53%	90.50%	90.50%
	H and E normalized	94.70%	94.69%	94.70%	94.69%
	Macenko colour normalized	97.95%	97.96%	97.95%	97.95%
	Vahadane colour normalized	98.80%	98.80%	98.80%	98.80%
AdaBoost	H separated	76.35%	77.09%	76.35%	76.03%
	E separated	82.15%	82.03%	82.15%	82.07%
	H and E normalized	84.75%	84.76%	84.75%	84.67%
	Macenko colour normalized	90.25%	90.35%	90.25%	90.22%
	Vahadane colour normalized	91.15%	91.57%	91.15%	91.13%
Decision tree	H separated	84.20%	84.21%	84.20%	84.20%
	E separated	79.40%	79.52%	79.40%	79.43%
	H and E normalized	86.45%	86.54%	86.45%	86.46%
	Macenko colour normalized	93.45%	93.45%	93.45%	93.45%
	Vahadane colour normalized	93.70%	93.71%	93.70%	93.70%
Naïve Bayes	H separated	76.65%	77.74%	76.65%	76.55%
	E separated	65.00%	67.77%	65.00%	64.68%
	H and E normalized	77.40%	78.66%	77.40%	77.26%
	Macenko colour normalized	84.85%	85.23%	84.85%	84.82%
	Vahadane colour normalized	88.50%	88.63%	88.50%	88.54%

**TABLE 8:** Complete performance comparison of all models across different normalization applied for dataset‐2.

Model	Normalization method	Accuracy	Precision	Recall	F1 score
Stacking model	H separated	99.13%	99.13%	99.13%	99.12%
	E separated	93.63%	93.63%	93.63%	93.63%
	H and E normalized	96.00%	96.08%	96.00%	96.02%
	Macenko colour normalized	99.13%	99.12%	99.13%	99.12%
	Vahadane colour normalized	99.25%	99.25%	99.25%	99.25%
MLP	H separated	98.25%	98.25%	98.25%	98.25%
	E separated	92.38%	92.32%	92.38%	92.34%
	H and E normalized	93.63%	93.80%	93.63%	93.67%
	Macenko colour normalized	99.13%	99.12%	99.13%	99.12%
	Vahadane colour normalized	99.20%	99.20%	99.20%	99.20%
LGBM	H separated	98.13%	98.17%	98.13%	98.12%
	E separated	92.50%	92.66%	92.50%	92.54%
	H and E normalized	93.63%	93.80%	93.63%	93.68%
	Macenko colour normalized	98.00%	98.00%	98.00%	98.00%
	Vahadane colour normalized	98.63%	98.63%	98.63%	98.63%
XGB	H separated	97.63%	97.68%	97.63%	97.63%
	E separated	91.00%	91.23%	91.00%	91.06%
	H and E normalized	92.00%	92.19%	92.00%	92.04%
	Macenko colour normalized	97.88%	97.87%	97.88%	97.87%
	Vahadane colour normalized	98.00%	98.05%	98.00%	98.01%
KNN	H separated	97.88%	97.89%	97.88%	97.88%
	E separated	90.38%	90.47%	90.38%	90.40%
	H and E normalized	92.63%	92.71%	92.63%	92.65%
	Macenko colour normalized	97.13%	97.14%	97.13%	97.12%
	Vahadane colour normalized	97.88%	97.89%	97.88%	97.88%
SVM	H separated	96.75%	96.83%	96.75%	96.76%
	E separated	84.38%	85.04%	84.38%	84.54%
	H and E normalized	89.50%	89.57%	89.50%	89.49%
	Macenko colour normalized	96.75%	96.75%	96.75%	96.74%
	Vahadane colour normalized	97.63%	97.66%	97.63%	97.63%
Extra trees	H separated	97.63%	97.68%	97.63%	97.63%
	E separated	90.38%	90.65%	90.38%	90.42%
	H and E normalized	92.13%	92.27%	92.13%	92.17%
	Macenko colour normalized	96.75%	96.75%	96.75%	96.75%
	Vahadane colour normalized	97.38%	97.39%	97.38%	97.38%
Random forest	H separated	96.88%	96.93%	96.88%	96.88%
	E separated	88.88%	89.22%	88.88%	88.97%
	H and E normalized	91.13%	91.26%	91.13%	91.18%
	Macenko colour normalized	96.63%	96.62%	96.63%	96.62%
	Vahadane colour normalized	97.00%	97.02%	97.00%	97.01%
Gradient boosting	H separated	96.75%	96.81%	96.75%	96.76%
	E separated	87.13%	87.30%	87.13%	87.17%
	H and E normalized	90.00%	90.16%	90.00%	90.06%
	Macenko colour normalized	96.75%	96.75%	96.75%	96.75%
	Vahadane colour normalized	96.75%	96.83%	96.75%	96.77%
Logistic regression	H separated	95.00%	95.11%	95.00%	95.03%
	E separated	82.13%	82.63%	82.13%	82.25%
	H and E normalized	88.13%	88.16%	88.13%	88.12%
	Macenko colour normalized	95.25%	95.25%	95.25%	95.23%
	Vahadane colour normalized	96.88%	96.90%	96.88%	96.88%
AdaBoost	H separated	77.75%	77.71%	77.75%	77.70%
	E separated	75.88%	75.71%	75.88%	75.70%
	H and E normalized	77.75%	77.72%	77.75%	77.68%
	Macenko colour normalized	88.38%	88.31%	88.38%	88.33%
	Vahadane colour normalized	86.63%	86.58%	86.63%	86.51%
Decision tree	H separated	78.13%	79.62%	78.13%	78.58%
	E separated	76.38%	77.26%	76.38%	76.47%
	H and E normalized	78.13%	79.62%	78.13%	78.58%
	Macenko colour normalized	89.00%	89.02%	89.00%	89.00%
	Vahadane colour normalized	90.88%	90.87%	90.88%	90.86%
Naïve Bayes	H separated	71.00%	73.35%	71.00%	70.85%
	E separated	66.75%	68.15%	66.75%	66.47%
	H and E normalized	71.00%	73.35%	71.00%	70.85%
	Macenko colour normalized	79.25%	80.05%	79.25%	78.88%
	Vahadane colour normalized	83.13%	83.21%	83.13%	82.99%

**TABLE 9:** Complete performance comparison of all models across different normalization applied for merged dataset.

Model	Normalization method	Accuracy	Precision	Recall	F1 score
Stacking model	H separated	98.29%	98.30%	98.29%	98.29%
	E separated	95.96%	95.98%	95.96%	95.96%
	H and E normalized	97.78%	97.78%	97.78%	97.78%
	Macenko colour normalized	99.58%	99.58%	99.58%	99.58%
	Vahadane colour normalized	99.33%	99.34%	99.33%	99.33%
MLP	H separated	97.63%	97.63%	97.63%	97.63%
	E separated	95.33%	95.33%	95.33%	95.33%
	H and E normalized	96.29%	96.32%	96.29%	96.30%
	Macenko colour normalized	99.54%	99.54%	99.54%	99.54%
	Vahadane colour normalized	99.25%	99.25%	99.25%	99.25%
LGBM	H separated	96.54%	96.57%	96.54%	96.55%
	E separated	93.58%	93.57%	93.58%	93.57%
	H and E normalized	96.42%	96.43%	96.42%	96.41%
	Macenko colour normalized	98.67%	98.68%	98.67%	98.67%
	Vahadane colour normalized	98.92%	98.92%	98.92%	98.92%
XGB	H separated	95.50%	95.53%	95.50%	95.51%
	E separated	92.25%	92.22%	92.25%	92.23%
	H and E normalized	95.47%	95.49%	95.47%	95.46%
	Macenko colour normalized	98.33%	98.35%	98.33%	98.34%
	Vahadane colour normalized	98.58%	98.59%	98.58%	98.59%
KNN	H separated	95.88%	95.94%	95.88%	95.89%
	E separated	93.13%	93.12%	93.13%	93.10%
	H and E normalized	96.17%	96.18%	96.17%	96.17%
	Macenko colour normalized	98.79%	98.80%	98.79%	98.79%
	Vahadane colour normalized	98.67%	98.68%	98.67%	98.67%
SVM	H separated	93.54%	93.62%	93.54%	93.57%
	E separated	90.38%	90.33%	90.38%	90.34%
	H and E normalized	93.95%	93.99%	93.95%	93.93%
	Macenko colour normalized	98.04%	98.05%	98.04%	98.04%
	Vahadane colour normalized	98.21%	98.23%	98.21%	98.21%
Extra trees	H separated	93.50%	93.58%	93.50%	93.52%
	E separated	91.13%	91.10%	91.13%	91.09%
	H and E normalized	94.03%	94.16%	94.03%	94.01%
	Macenko colour normalized	97.25%	97.29%	97.25%	97.26%
	Vahadane colour normalized	98.00%	98.01%	98.00%	98.00%
Random forest	H separated	94.08%	94.14%	94.08%	94.10%
	E separated	90.46%	90.42%	90.46%	90.43%
	H and E normalized	93.25%	93.32%	93.25%	93.22%
	Macenko colour normalized	97.29%	97.32%	97.29%	97.30%
	Vahadane colour normalized	98.17%	98.18%	98.17%	98.17%
Gradient boosting	H separated	92.46%	92.49%	92.46%	92.46%
	E separated	89.58%	89.56%	89.58%	89.55%
	H and E normalized	92.63%	92.67%	92.63%	92.61%
	Macenko colour normalized	97.83%	97.85%	97.83%	97.84%
	Vahadane colour normalized	97.50%	97.54%	97.50%	97.51%
Logistic regression	H separated	92.46%	92.51%	92.46%	92.48%
	E separated	89.42%	89.35%	89.42%	89.37%
	H and E normalized	93.04%	93.06%	93.04%	93.01%
	Macenko colour normalized	97.63%	97.63%	97.63%	97.63%
	Vahadane colour normalized	97.58%	97.60%	97.58%	97.59%
AdaBoost	H separated	77.96%	78.77%	77.96%	77.89%
	E separated	79.00%	78.89%	79.00%	78.93%
	H and E normalized	81.84%	81.96%	81.84%	81.65%
	Macenko colour normalized	87.58%	87.62%	87.58%	87.47%
	Vahadane colour normalized	89.00%	89.75%	89.00%	88.73%
Decision tree	H separated	85.42%	85.62%	85.42%	85.47%
	E separated	75.92%	76.48%	75.92%	76.10%
	H and E normalized	83.61%	83.65%	83.61%	83.63%
	Macenko colour normalized	91.92%	91.97%	91.92%	91.93%
	Vahadane colour normalized	92.33%	92.45%	92.33%	92.36%
Naïve Bayes	H separated	75.33%	76.26%	75.33%	74.90%
	E separated	64.46%	67.59%	64.46%	64.18%
	H and E normalized	74.02%	75.22%	74.02%	73.95%
	Macenko colour normalized	82.88%	83.45%	82.88%	82.82%
	Vahadane colour normalized	88.25%	88.41%	88.25%	88.28%

The performance metrics of various models across different normalization method is detailed in Table 7 for dataset 1, reveal significant insights into the efficacy of different stain normalization techniques and classifiers in histopathology image analysis.

Analysing the impact of stain normalization methods, it's evident that the Vahadane colour normalized datasets consistently lead to superior model performance. For instance, the stacking model achieved an accuracy of 99.95% on the Vahadane normalized data, slightly outperforming its performance on the Macenko colour normalized data set, which was 99.85%. This trend is consistent across other classifiers; the MLP model reached an accuracy of 99.90% with Vahadane normalization, compared to 99.75% with Macenko normalization. The KNN classifier also demonstrated enhanced performance on Vahadane normalized data, achieving 99.75% accuracy, whereas it recorded 99.20% on Macenko normalized data. These observations suggest that the Vahadane normalization technique may provide a more robust framework for reducing colour‐induced variability in histopathology images, thereby facilitating improved classifier performance.

In contrast, datasets processed with H separated, E separated, and H&E normalized techniques yielded comparatively lower performance metrics across all models. The proposed stacking model, for example, recorded accuracies of 98.10%, 97.10% and 98.25% on H separated, E separated and H&E normalized datasets, respectively. Similarly, the MLP model achieved accuracies of 97.35% on H separated data, 95.70% on E separated data and 97.70% on H&E normalized data. The KNN classifier followed this pattern, with accuracies of 95.95%, 94.15% and 96.75% on H separated, E separated and H&E normalized datasets, respectively. These results indicate that while H and E separation techniques contribute to model performance, the inclusion of H&E normalization leads to slight improvements. However, they may still not be as effective as comprehensive normalization methods like Vahadane and Macenko in standardizing stain variations and enhancing classification accuracy.

The empirical evidence presented in Table 7 for dataset 1, highlights the critical influence of stain normalization techniques and classifier selection on model performance. The Vahadane normalization method, in conjunction with the Stacking classifier, emerges as a highly effective approach, offering significant improvements in the accuracy and reliability of histopathological image analysis.

**FIGURE 12:**
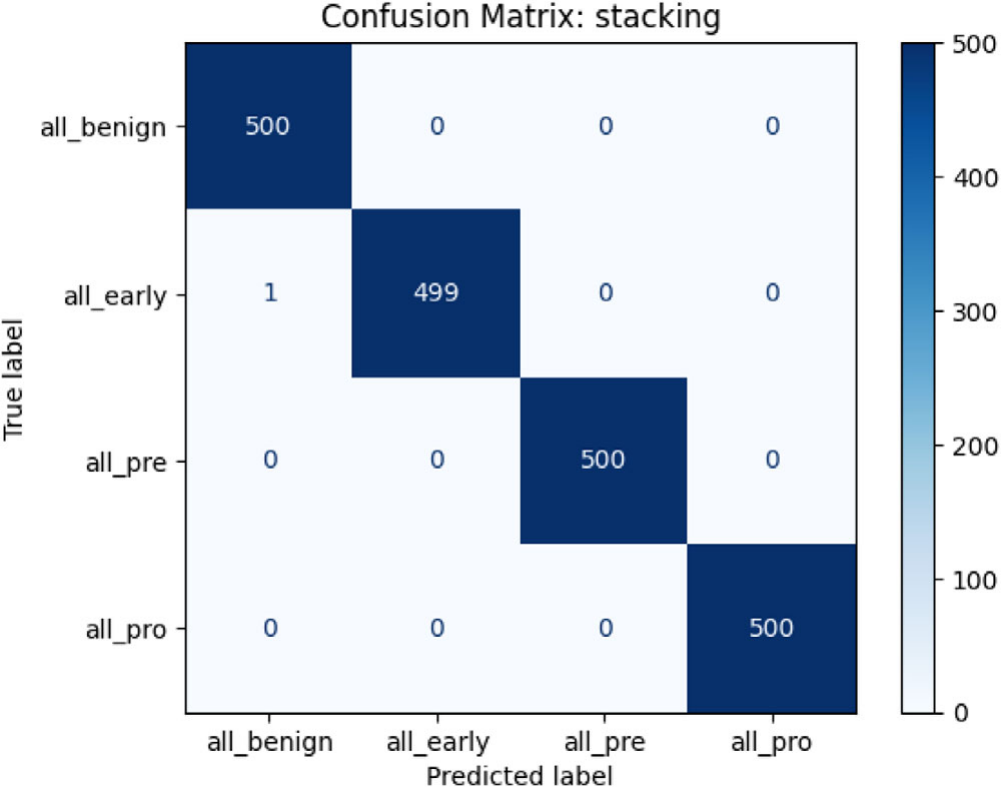
Confusion matrix for stacking ensemble classifier for first dataset “dataset 1” with Vahadane colour normalization.

Out of the 2000 test images in dataset 1, only one instance of the “malignant early” subtype was misclassified as “benign” as shown in the confusion matrix for the stacking classifier in the Vahadane colour normalization dataset in Figure 12. This single miss‐classification represents an error rate of merely 0.05%, underscoring the ensemble model and Vahadane colour normalization's robustness. The model displayed perfect performance for all other categories, achieving 100% precision, recall, and F1‐scores for the “Pre‐B” and “Pro‐B” malignant subtypes. Due to the balanced class distribution, this single misclassification affects all evaluation metrics uniformly, resulting in nearly identical values for accuracy, precision, recall, and F1‐score. The 95% confidence interval for accuracy is [99.85%, 100%], further supporting the reliability of the reported performance.

To evaluate the broader applicability and robustness of the suggested methodology, a second dataset “dataset 2”, initially consisting of 3252 images, was utilized. This dataset provided a challenging scenario to test the robustness of the classifiers due to its limited data volume. To mitigate potential issues related to data scarcity and enhance model generalization, the dataset was augmented to 8000 images. Despite this augmentation, the overall ranking of model performance remained consistent, with the Vahadane colour normalization method and proposed stacking model maintaining their position as the best‐performing method. The performance metrics for all models on the augmented second dataset “dataset 2” are summarized in Table 8, demonstrating that even with artificially expanded data, the relative effectiveness of the classifiers remained unchanged, further reinforcing the superiority and robustness of the Stacking model in this classification task.

**FIGURE 13:**
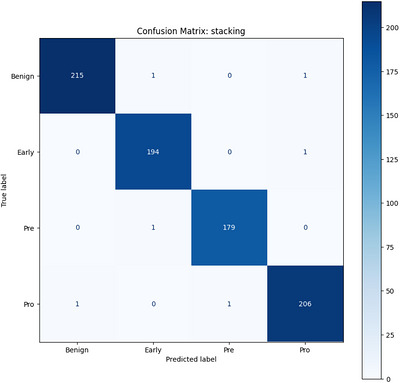
Confusion matrix for stacking ensemble classifier for second dataset “dataset 2” with Vahadane colour normalization.

While the performance metrics for the second dataset were slightly lower compared to the primary dataset, the overall trend remained consistent as shown in Table 8. The ensemble model achieved 99.25% accuracy, maintaining its superiority. A few models, such as decision yree, and naïve Bayes, Adaboost experienced a more significant drop in performance, particularly due to their sensitivity to data distribution and volume. The confusion matrix in Figure 13 for the stacking classifier on the second dataset shows strong performance across all categories, with very few miss‐classifications. Additionally, the 95% confidence interval for the Stacking model with Vahadane normalization on dataset‐2 is [98.83%, 99.67%], confirming statistically reliable performance. This indicates the model's ability to generalize well, even with fewer data.

**FIGURE 14:**
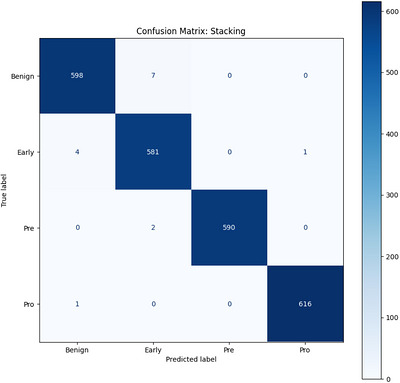
Confusion matrix for stacking ensemble classifier for merged dataset with Vahadane colour normalization.

Furthermore, when the datasets were merged, combining dataset‐1 and dataset‐2, the overall performance further improved from dataset‐2. As shown in Table 9, the ensemble model achieved an accuracy of 99.33%, with Vahadane colour normalization, demonstrating enhanced generalization with a larger dataset. The confusion matrix in Figure 14 highlights the model's robustness across all categories, with minimal misclassifications. This suggests that even after integrating both datasets, the model's predictive capabilities remains superior.

One notable observation from the merged dataset results (Table 9) is that Macenko normalization achieved a slightly higher stacking accuracy of 99.58%, compared to 99.33% with Vahadane normalization, corresponding to a difference of 0.25 percentage points. This is the only case across all three datasets where Macenko outperforms Vahadane. A likely contributing factor is the heterogeneous nature of the merged dataset, which combines images from multiple sources with varying acquisition conditions and staining protocols. Under such cross‐domain variability, Macenko's SVD‐based stain estimation may better capture global stain characteristics, whereas Vahadane's NMF‐based method, although highly effective for within‐domain normalization, may be less robust to broader distribution shifts. Despite this, Vahadane demonstrates consistently strong and competitive performance across all datasets and classifiers, supporting its selection as the preferred normalization strategy. Future work should explore adaptive, dataset‐specific normalization techniques within automated preprocessing pipelines.

To further confirm the stability of the proposed stacking ensemble with Vahadane normalization, a 5‐fold cross‐validation was performed on the merged dataset. In each fold, the dataset was partitioned such that 80% of the data was used for training and the remaining 20% for validation, with augmentation applied exclusively within each training fold to prevent any form of data leakage across folds. Due to computational limitations, this validation was restricted to the best‐performing preprocessing and classifier combination only. The fold‐wise results are presented in Table 10.

**TABLE 10:** 5‐fold cross‐validation results for stacking ensemble classifier with Vahadane colour normalization on merged dataset.

Fold	Accuracy	Precision	Recall	F1‐score
Fold 1	99.21%	99.22%	99.21%	99.21%
Fold 2	99.33%	99.33%	99.33%	99.33%
Fold 3	99.18%	99.19%	99.18%	99.18%
Fold 4	99.35%	99.35%	99.35%	99.35%
Fold 5	99.29%	99.29%	99.29%	99.29%
Mean	99.27%	99.28%	99.27%	99.27%
Std Dev	±0.07%	±0.07%	±0.07%	±0.07%

The model achieved a mean accuracy of 99.27% ± 0.07% with a 95% confidence interval of [99.18%, 99.36%], which falls slightly below but remains closely aligned with the 99.33% accuracy observed during independent hold‐out testing. The observed difference between cross‐validation and hold‐out results can be attributed to variations in data partitioning across evaluation strategies. The narrow confidence interval and consistently low variance across folds indicate stable performance and strong generalization across different data subsets.

The evaluation using three datasets demonstrates how reliable and flexible our proposed methodology is. The colour normalization using Vahadane method along with the ensemble model, particularly our proposed stacking classifier, consistently delivered high performance with top‐tier metrics in accuracy, recall, precision and F1‐score, confirming the effectiveness of combining predictions from multiple classifiers. Even with a smaller dataset (dataset‐2), the model maintained an impressive accuracy which is 99.25%, demonstrating its generalizability and scalability under constrained conditions. While models like MLP, SVM and XGB showed resilience, simpler models such as decision tree and naïve Bayes struggled significantly, underscoring the importance of advanced ensemble techniques for achieving reliable and robust performance. Moreover, since our approach leverages pretrained deep learning features instead of retraining models from scratch, it substantially reduces computational cost and training time while preserving high accuracy and generalization capability.

Using PBS pictures, the suggested methodology focuses on effortlessly identifying and classifying ALL, B‐ALL and its subgroups. However, this study has certain limitations. The primary limitation is the reliance on datasets collected from a single source, which might constrain the model's generalizability across diverse patient populations. Even though the two datasets included in this study are large, they might not accurately reflect the variability observed in actual clinical settings, which could have an impact on how well the model performs when applied to data that the model hasn't seen before. Additionally, the current approach evaluates only one diagnostic modality–peripheral blood smear images, while an accurate diagnosis in clinical practice often requires integrating multiple diagnostic techniques such as bone marrow cytomorphology, flow cytometry and genetic profiling. Expanding the framework to incorporate these modalities could improve the robustness of machine learning models in aiding clinical decision‐making.

Additionally, the methodology proposed here is tailored specifically for ALL, B‐ALL and its subtypes, and its direct applicability to other hematological disorders or medical conditions remains uncertain. Even though class imbalance in the second dataset was addressed using augmentation, the dataset's fundamental features might still restrict the model's applicability in other medical fields.

Finally, although the Vahadane colour normalization combined with the stacking ensemble approach demonstrated strong performance, the absence of external validation on independent datasets limits its generalizability. In particular, the lack of evaluation on data from different institutions or geographic regions means the findings should be interpreted as proof of concept rather than evidence of clinical readiness. While the near‐perfect performance metrics are encouraging, they require confirmation through prospective and externally validated studies. Future work should address these limitations by incorporating multi‐institutional datasets and exploring the integration of multi‐modal diagnostic information to enhance the robustness and clinical applicability of the proposed system.

### 4.2 Explainable AI Visualizations

**FIGURE 15:**
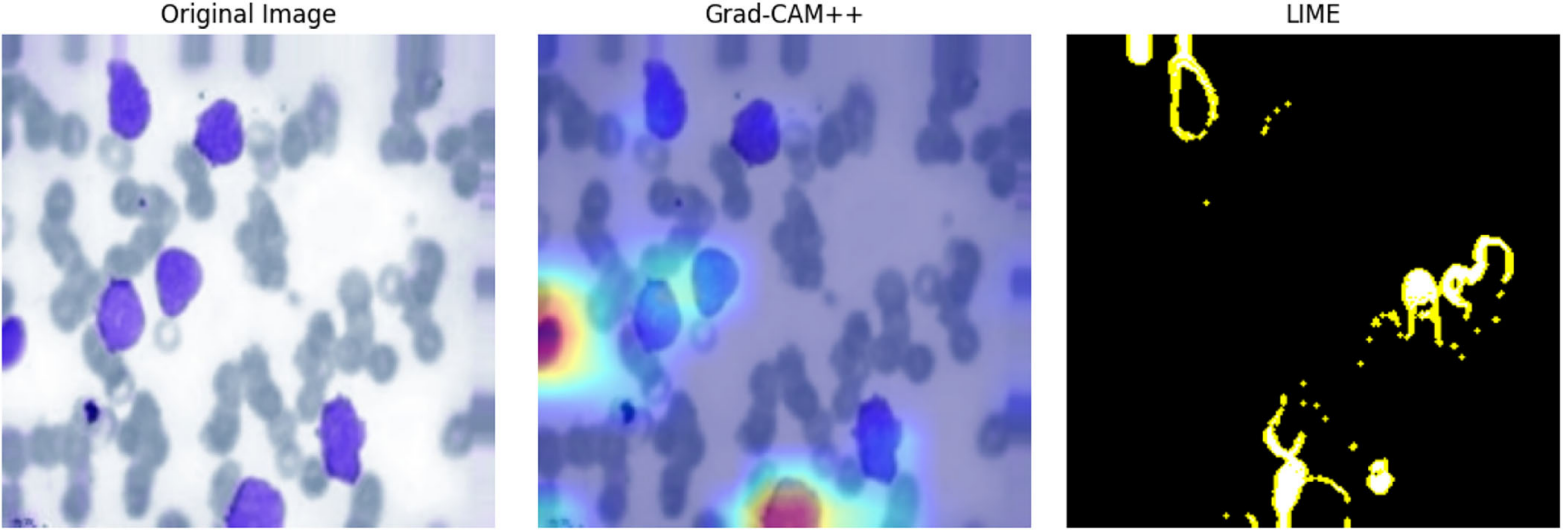
Grad‐CAM++ and LIME visualization for dataset‐1.

**FIGURE 16:**
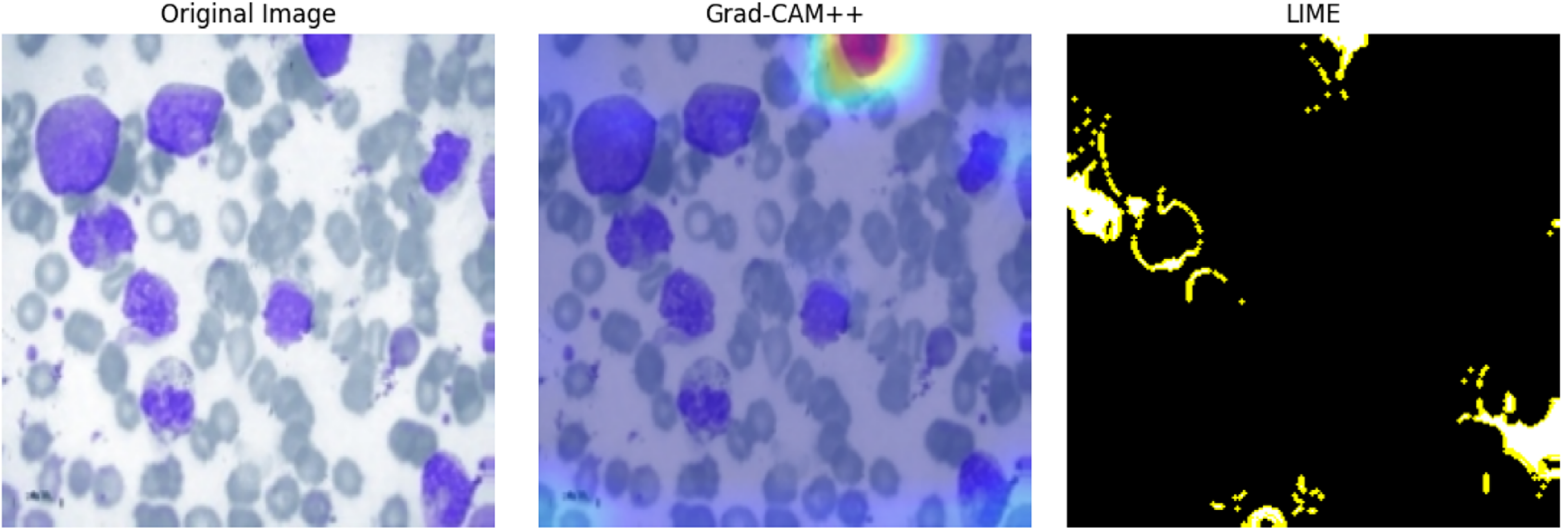
Grad‐CAM++ and LIME visualization for dataset‐2.

**FIGURE 17:**
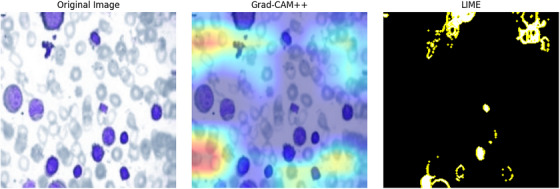
Grad‐CAM++ and LIME visualization for merged dataset.

Figures 15, 16, 17 present the Grad‐CAM++ and LIME visualization, which emphasizes the important features relevant to the prediction. In general, these results highlight the effectiveness of our proposed model for classifying ALL, B‐ALL and its subtype.

### 4.3 Medical Application and Real‐Time Classification Result

**FIGURE 18:**
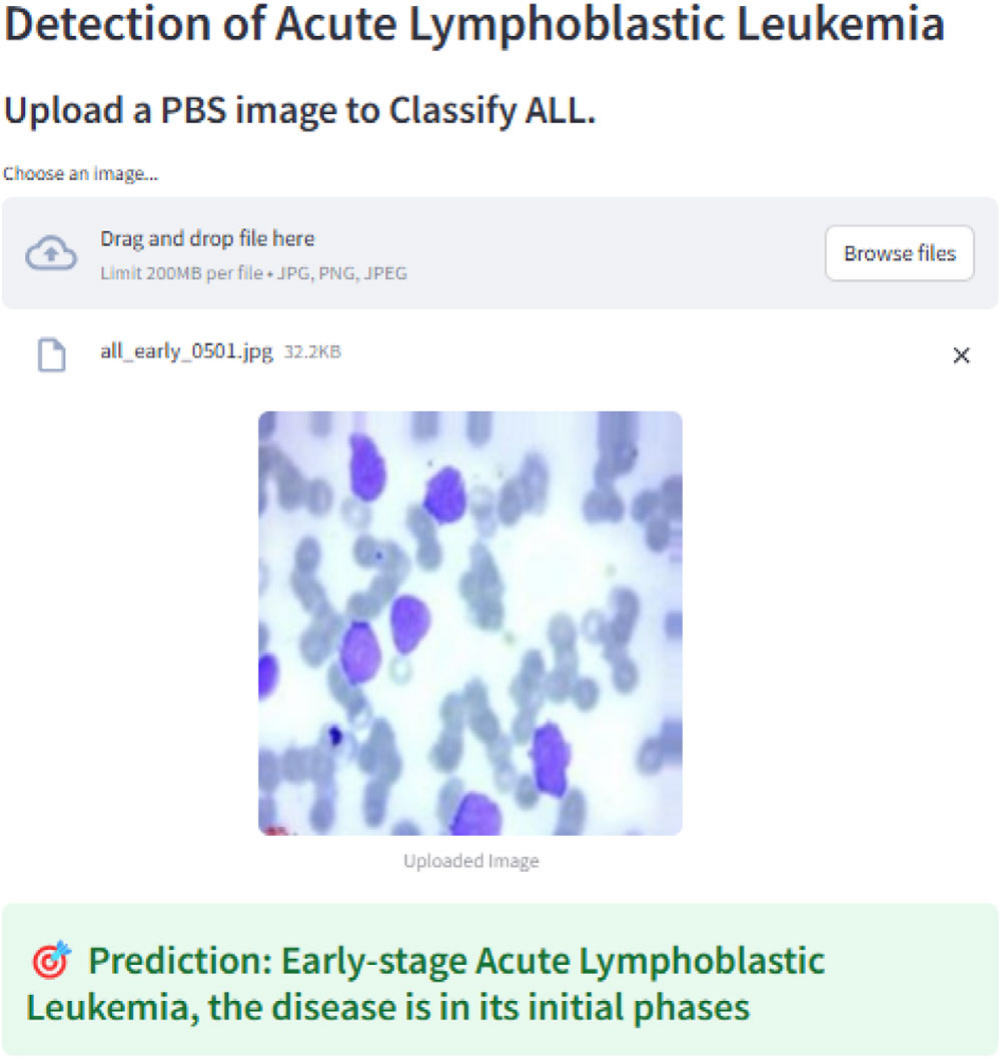
Proposed medical applications output.

The website demonstration for a PBS image in Figure 18, where the model predicts the class along with an explanation and confidence score. The user uploads a PBS image, and the system classifies it as early‐stage ALL, indicating that the disease is in its initial phases. The model provides a confidence score of 99.90%, reflecting its high certainty and reliability in classifying ALL from PBS images.

### 4.4 Discussion

We have compared our work with existing work and see that our proposed model performs superior. The following Table 11 mentions the comparison between our proposed method and existing research.

**TABLE 11:** Comparison of proposed performance with existing research.

Authors	Dataset	Proposed Models	Accuracy	XAI Integration	Real Time Classification
Sampathila et al. (2022) [3]	ALL challenge dataset from ISBI 2019	ALLNET (Customized CNN)	95.54%	N/A	N/A
Jha and Dutta (2019) [15]	AA‐IDB2 database (blood smear images)	Hybrid model using mutual information, fuzzy C‐means, active contour and CNN with chronological SCA	98.7%	N/A	N/A
Khandekar et al. (2021) [17]	C_NMC_2019 and ALL‐IDB1	YOLO v4 object detection algorithm	98.7% (C_NMC_2019), 96.06% (ALL‐IDB1)	N/A	N/A
Ghaderzadeh et al. (2022) [18]	Acute lymphoblastic leukaemia (ALL) image dataset(2021) from Kaggle	DenseNet‐201 with cost‐conscious cell segmentation	99.85%	N/A	N/A
Eckardt et al. (2022) [19]	Dataset of 1487 AML cases and 236 healthy controls	Multi‐step deep neural networks with Faster R‐CNN	87.3% (AML detection), 86% (NPM1 mutation status prediction)	N/A	N/A
Mondal et al. (2021) [20]	CNMC‐2019	Ensemble of InceptionResNet‐V2, MobileNetV2, VGG‐16, Xception, DenseNet‐121	86.2%	N/A	N/A
Jiwani et al. (2023) [1]	Not specified	ALLDM model analysing white blood cell count data	Chemotherapy: 81.53% (DDS) and 87.92% (SDS) Stem Cell Transplantation: 79.16% (DDS) and 94.31% (SDS) Radiation Therapy: 63.77% (DDS) and 87.37% (SDS) Targeted Therapy: 88.92% (DDS) and 85.86% (SDS)	N/A	N/A
**Proposed**	Dataset A	Stacking ensemble model	99.95%	Grad‐CAM++ and LIME	Yes
**Proposed**	Dataset B	Stacking ensemble model	99.25%	Grad‐CAM++ and LIME	Yes
**Proposed**	Merged dataset	Stacking ensemble model	99.33%	Grad‐CAM++ and LIME	Yes

## 5 Conclusion

In summary, this research introduces an innovative method for the prompt and precise identification of ALL and its various subtypes through the use of stain normalization and a hybrid ensemble system. By leveraging the colour normalization using Vahadane method with the feature extraction capabilities of VGG16 and integrating various machine learning models into a stacking ensemble classifier, the proposed system achieves exceptional performance metrics, including 99.95% accuracy, recall, precision and F1‐score. This demonstrates the platform's effectiveness in distinguishing ALL cases from benign hematogones and accurately classifying its subtypes, addressing a critical gap in traditional diagnostic methods.

The proposed platform leverages non‐invasive PBS images, reducing reliance on more invasive and costly diagnostic procedures, and serving as a promising proof of concept for future clinical decision‐support systems, subject to external validation on geographically and institutionally diverse datasets. Its consistently high recall supports reliable identification of true positive cases, thereby reducing the likelihood of missed diagnoses. Such performance may assist oncologists in treatment planning and decision‐making, with the potential to improve patient outcomes following further clinical validation.

Even with its encouraging outcomes, the platform's performance has to be further tested on a variety of datasets and in actual clinical settings to guarantee its generalizability. Future research endeavours will concentrate on augmenting the system's resilience through the integration of hybrid deep learning methodologies, such as merging convolutional and recurrent neural networks, and exploring its applicability for identifying different hematological abnormalities.

Overall, this study demonstrates the potential of integrating deep learning and machine learning ensemble techniques along with stain normalization in medical diagnostics, paving the way for more accessible, accurate and efficient tools to assist clinicians in the fight against life‐threatening conditions like ALL.

## Author Contributions

**Faysal Ahmmed**: conceptualization, data curation, formal analysis, methodology, software, visualization, writing – original draft. **Md. Sadi Al Huda**: investigation, methodology, project administration, writing – review & editing. **Md. Asraf Ali**: supervision, validation, writing – review & editing. **M. M. Manjurul Islam**: project administration, supervision, validation, writing – review & editing. All authors have read and agreed to the published version of the manuscript.

## Funding

The authors have nothing to report.

## Conflicts of Interest

The authors declare no conflicts of interest.

## Data Availability

The data used in this study are publicly available from Kaggle: https://www.kaggle.com/datasets/mohammadamireshraghi/blood‐cell‐cancer‐all‐4class.
